# Transcriptional profiling of pea *ABR17 *mediated changes in gene expression in *Arabidopsis thaliana*

**DOI:** 10.1186/1471-2229-8-91

**Published:** 2008-09-10

**Authors:** Sowmya S Krishnaswamy, Sanjeeva Srivastava, Mohsen Mohammadi, Muhammad H Rahman, Michael K Deyholos, Nat NV Kav

**Affiliations:** 1 Department of Agricultural, Food and Nutritional Science, University of Alberta, Edmonton, AB T6G 2P5, Canada; 2 Department of Biological Sciences, University of Alberta, Edmonton, AB T6G 2E9, Canada

## Abstract

**Background:**

Pathogenesis-related proteins belonging to group 10 (PR10) are elevated in response to biotic and abiotic stresses in plants. Previously, we have shown a drastic salinity-induced increase in the levels of ABR17, a member of the PR10 family, in pea. Furthermore, we have also demonstrated that the constitutive expression of pea *ABR17 *cDNA in *Arabidopsis thaliana *and *Brassica napus *enhances their germination and early seedling growth under stress. Although it has been reported that several members of the PR10 family including ABR17 possess RNase activity, the exact mechanism by which the aforementioned characteristics are conferred by ABR17 is unknown at this time. We hypothesized that a study of differences in transcriptome between wild type (WT) and *ABR17 *transgenic *A. thaliana *may shed light on this process.

**Results:**

The molecular changes brought about by the expression of pea *ABR17 *cDNA in *A. thaliana *in the presence or absence of salt stress were investigated using microarrays consisting of 70-mer oligonucleotide probes representing 23,686 *Arabidopsis *genes. Statistical analysis identified number of genes which were over represented among up- or down-regulated transcripts in the transgenic line. Our results highlight the important roles of many abscisic acid (ABA) and cytokinin (CK) responsive genes in *ABR17 *transgenic lines. Although the transcriptional changes followed a general salt response theme in both WT and transgenic seedlings under salt stress, many genes exhibited differential expression patterns when the transgenic and WT lines were compared. These genes include plant defensins, heat shock proteins, other defense related genes, and several transcriptional factors. Our microarray results for selected genes were validated using quantitative real-time PCR.

**Conclusion:**

Transcriptional analysis in *ABR17 *transgenic *Arabidopsis *plants, both under normal and saline conditions, revealed significant changes in abundance of transcripts for many stress responsive genes, as well as those related to plant growth and development. Our results also suggest that *ABR17 *may mediate stress tolerance through the modulation of many ABA- and CK-responsive genes and may further our understanding of the role of ABR17 in mediating plant stress responses.

## Background

Pathogenesis-related (PR) proteins are part of the plant defense responses that are induced by pathogens as well as by abiotic stresses [1,2]. To date, 17 different families of PR proteins have been identified, based on their specific structural and functional properties [[3] and references therein]. Among the PR proteins, the PR10 family is composed of intracellular acidic proteins with molecular masses ranging from 15–18 kD and are encoded by multiple genes [1,3]. *PR10 *genes were first described in *Pisum sativum *inoculated with *Fusarium solani *[4] but have been subsequently described in many species [reviewed in [3]]. In addition to their inducible expression in response to stresses, *PR10 *genes also exhibit constitutive high expressed levels in roots, flowers and pollen during normal growth and development, suggesting additional roles beyond pathogenesis responses [5].

Based on sequence similarities, PR10 proteins have been suggested to be ribonucleases (RNases) [6]. Indeed, PR10 proteins from a variety of species including two pea PR10 proteins have been demonstrated to possess RNase activity [7,8]. Although RNase activities have been detected for many PR10 proteins, they have also been shown to interact with molecules such as cytokinins (CKs), brassinosteroids, fatty acids, and flavonoids [9-11]. These observations have led to the suggestion that all PR10 proteins may not be RNases and may be involved during normal plant growth and development as hormone/ligand carriers [10-12]. This suggestion is further supported by the fact that CK-specific binding proteins (CSBPs) exhibit amino acid sequence and predicted secondary-structure similarities with PR10 proteins and, for this reason, have been included in the PR10 family [9].

The pea abscisic acid-responsive protein ABR17, induced by the exogenous application of abscisic acid (ABA) is classified as a member of the PR10 family in pea [13]. ABR17 is produced late in seed development, and is homologous to dehydrins and late embryogenesis abundant (LEA)-related proteins [14,15]. ABR17 is also significantly homologous to intracellular pathogenesis related (IPR) proteins and major birch pollen allergen Betv1 proteins [16,17]. Our previous research has demonstrated the expression of ABR17 protein in pea under salt stress [2] and the RNase activity of two members of pea PR10 proteins (PR10.1 and ABR17) [7,8]. Furthermore, we have also demonstrated that the constitutive expression of pea *PR10.1 *and *ABR17 *cDNAs enhance germination and early seedling growth under abiotic stress conditions in *B. napus *and *A. thaliana*, respectively [18,19]. In addition, the transgenic plants also exhibited phenotypic differences when compared to their WT counterparts, which included precocious flowering, a higher number of lateral branches, and increased numbers of seed pods [8]. Many of these characteristics of *ABR17*-transgenic *A. thaliana *are suggestive of a role for CKs in ABR17 action, particularly increased lateral branching and early flowering [20,21]. Our suggestion was further supported by the elevated concentrations of endogenous CKs in *PR10.1 *transgenic *B. napus *as well as *ABR17*-transgenic *A. thaliana *[7,8].

These observations led us to hypothesize that PR10 proteins, including ABR17, may mediate the observed phenotypic effects through modulation of endogenous CKs. Additional evidence supporting this hypothesis has been provided by the demonstration that exogenous application of CK enhances germination under abiotic stress conditions [8]. In order to further investigate the *ABR17*-mediated changes in *A. thaliana *we investigated global changes in gene expression using microarrays. Microarray analysis was carried out in an *ABR17*-transgenic line compared to its WT, salt treated *ABR17*-transgenic line compared to untreated *ABR17*-transgenic line, and salt treated WT compared to untreated WT seedlings. Our current findings reveal that, even in the absence of stress, the expression of genes involved in plant growth and development are significantly (and approximately 2-fold) increased in the transgenic line. Salt treated *ABR17*-transgenic *A. thaliana *seedlings showed general salt response theme comparable to that of the WT counterpart used in this study. However, both the trend as well as the degree of changes in gene expression of many defense related genes including plant defensins and heat shock proteins was different providing additional insights into the possible ways in which ABR17 may mediate plant responses to stress.

## Results and discussion

### Characterization of ABR17-transgenic plants/seedlings

**Figure 1:**
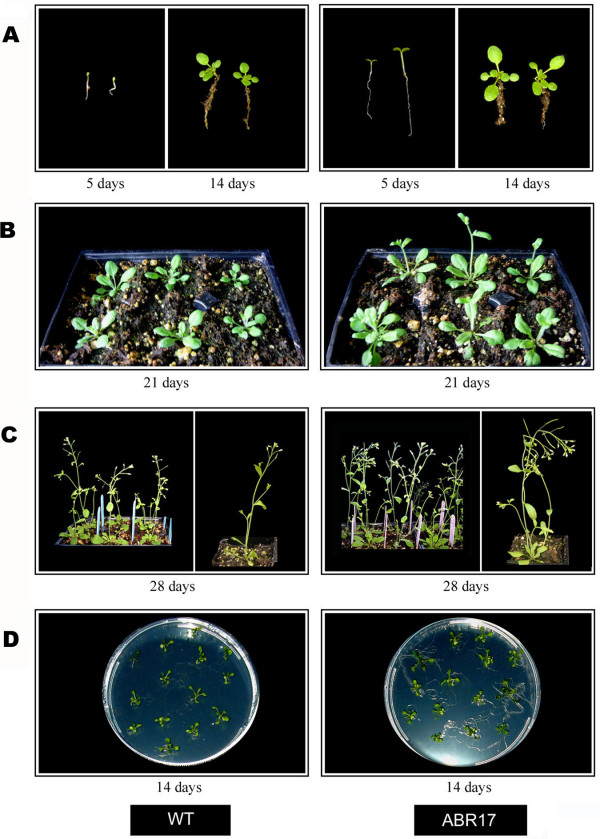
Appearance of WT and *ABR17 *transgenic *A. thaliana *at various growth stages: Seedlings at 5, 14 days (A), 21 days (B), 28 days (C) and MS-grown 14-day-old seedlings (D) are shown.

**Figure 2:**
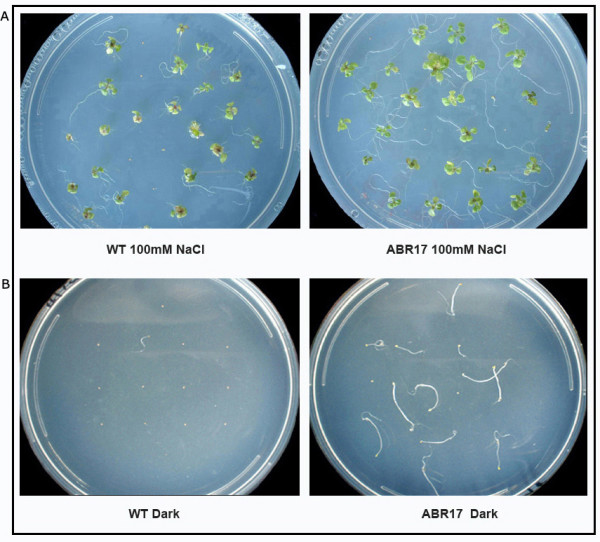
**Appearance of WT and *ABR17 *transgenic *A. thaliana *in response to treatments**. (A) Appearance of WT and transgenic *ABR17 A. thaliana *seedlings grown on MS media with 100 mM NaCl (B) Appearance of 7-day-old WT and *ABR17 *transgenic *A. thaliana *seedlings grown under dark.

The appearance of 2-week-old WT and *ABR17*- transgenic *A. thaliana *seedlings grown in soil as well as on MS medium (1.5% sucrose, 0.8% agar with pH 5.7) [22] plates are shown in Figure 1. At all growth stages investigated, the *ABR17*-transgenic line was considerably more developmentally advanced compared to its WT counterpart. For example, in the 5-day-old transgenic seedlings (Figure 1A) cotyledons were more developed than in their WT counterparts and at 14-days the transgenic seedlings possessed more rosette leaves (Figure 1A). Similar developmental differences were also observed at 21 days where many transgenic seedlings had started to bolt whereas very few (if any) WT seedlings had advanced to this developmental stage (Figure 1B). At 28 days, the transgenic seedlings also possessed more lateral branches (Figure 1C, Table 1). The transgenic seedlings also flowered earlier than WT with an average difference of at least 2.5 days (Table 1). Seedlings for microarray experiments were grown on semi-solid MS media in order to maintain sterility and it was evident that under these growth conditions also the transgenic seedlings were more developmentally advanced (Figure 1D). These results are consistent with our previous observations of seedlings from this and other independently derived *ABR17*-transgenic lines grown on semi solid MS media [19]. In addition, the *ABR17*-transgenic seedlings grown on MS media with 100 mM NaCl were greener and their roots appeared to be longer compared to the WT seedlings grown under similar conditions (Figure 2A).

**Table 1:** Morphological and physiological differences between WT and *ABR17*-transgenic *A. thaliana *lines

Morphological and pigment characteristics	WT	*ABR17*	p Value
	(Mean ± SE)	(Mean ± SE)	

Number of lateral branches (average)	3 ± 0.3	4.1 ± 0.2	0.025
Days to flower (average)	24 ± 0.1	21.6 ± 0.3	0.002
Germination in dark (Percent)	9.6 ± 3	84.4 ± 2	< 0.001
**Root length (cm)**			
0 mM NaCl	1.6 ± 0.1	2.3 ± 0.2	0.003
75 mM NaCl	0.7 ± 0.01	0.8 ± 0.02	0.012
100 mM NaCl	0.5 ± 0.03	0.6 ± 0.02	NS
**Fresh weight (g per 21 seedlings)**			
0 mM NaCl	0.10 ± 0.003	0.11 ± 0.005	NS
100 mM NaCl	0.014 ± 0.003	0.020 ± 0.0008	0.027
**Chlorophyll a/b (μg/g of FW)**			
0 mM NaCl	32.3 ± 1.26	33 ± 2.58	NS
100 mM NaCl	8 ± 0.93	13 ± 2.44	0.045
**Carotenoid (μg/g of FW)**			
0 mM NaCl	2.2 ± 0.063	2.3 ± 0.29	NS
100 mM NaCl	1.0 ± 0.207	1.4 ± 0.014	NS

NS: Non-significant

Characteristics like root length, fresh weight, chlorophyll a/b content and carotenoid contents were measured in salt treated- *ABR17 *and WT seedlings. Roots of *ABR17*-transgenic seedlings were relatively longer in the absence of salt whereas upon salt treatment, the differences in lengths were not that obvious (Table 1). The fresh weight of *ABR17*-transgenic seedlings was not significantly different from its WT counterpart in the absence of stress. However, in the presence of 100 mM NaCl, the fresh weights of the transgenic seedlings were significantly (p < 0.05) higher than their WT counterparts (Table 1). Although the chlorophyll and carotenoid contents were almost similar in *ABR17 *and WT seedlings without any stress, upon NaCl treatment the transgenic seedlings had significantly (p < 0.05) higher levels of chlorophyll (Table 1). Our results indicate that the NaCl treatment had less deleterious effects on the *ABR17*-transgenic seedlings compared to the WT.

In order to further characterize the differences between the WT and *ABR17*-transgenic lines, the ability of both WT and *ABR17*-transgenic seedlings to germinate in the presence or absence of light at RT was compared. In the dark, 85% of *ABR17*-transgenic *A. thaliana *had germinated after one week, whereas only 10% of the WT seeds had germinated under the same conditions (Table 1, Figure 2B). In contrast, in the presence of light, 100% of both *ABR17*-transgenic and WT seeds had germinated in the same period (data not shown). Most *Arabidopsis *ecotypes require light for germination, which is primarily controlled by a reversible red light dependent equilibrium of the photoreceptors [23]. It is also known that exogenous CKs can substitute for red light and enhance the germination of certain light-requiring species in the dark [24-27]. Furthermore, *A*. *thaliana *detiolated (*det*) mutants exhibits many characteristics of seeds germinated in the presence of light even when germinated in dark [28], a phenotype that has been attributed to CKs because of the fact that even WT seedlings exhibit the same phenotype when germinated in the dark following exogenous CK application [29]. A role for CKs can also be inferred from the observation that coumarin or far-red light, both of which prevent the formation of CK-nucleosides from storage forms of CKs, inhibit germination of lettuce seeds in the dark [30]. Interestingly, *amp1 A. thaliana *mutants, which possess higher endogenous CKs, also exhibited a photomorphogenic response that in part similar to our *ABR17*-transgenic seedlings [31]. Taken together, all these results seem to suggest that endogenous CKs play an important role in the germination of light-sensitive seeds and the elevated endogenous CKs in *ABR17*-transgenic seedlings previously reported [8] may be responsible for the enhanced germination of this genotype in the dark (Table 1, Figure 2B).

### Transcriptional profiling using microarrays

In order to characterize the molecular changes brought about by the expression of pea *ABR17 *cDNA in *A. thaliana *that resulted in the observed phenotypes, we analyzed gene expression by profiling the transcripts of *ABR17*-transgenic plants in the absence and presence of 100 mM NaCl. As described earlier, the first set of microarray analysis was the investigation of the differences in gene expression between *ABR17*-transgenic and WT *A. thaliana *in the absence of NaCl (*ABR17*/WT). The second set of microarray analysis was between 100 mM NaCl-treated WT and untreated WT *A. thaliana *(100 mM NaCl treated WT/WT). The third set of microarray analysis was between 100 mM NaCl treated *ABR17*-transgenic versus untreated *ABR17*-transgenic *A. thaliana *(100 mM NaCl treated *ABR17*/*ABR17*).

Microarrays (70-mer oligonucleotide microarrays) consisting of probes presenting 23,686 unique genes identified by *Arabidopsis *genome initiative (AGI) locus identifiers were used. We identified transcripts as those with mean signal intensities that differed significantly from 0 at α = 0.05 in a Student's *t*-test in each set of microarrays. The transcripts were categorized based on shared structural elements and/or inferred function. We selected 12 genes representing different functional categories, which according to our microarray analysis showed enhanced or reduced levels of transcript abundance to validate our microarrays. The results from microarrays and qRT-PCR analysis are discussed below.

### First set of transcriptional profiling: genes responsive to ABR17

Of the significantly responsive transcripts due the expression of pea ABR17 in *A. thaliana*, 124 were observed to be modulated in the transgenic line at least 1.5-fold compared to WT with 83 increasing and 41 decreasing in transcript abundance (Additional file 1). Many of these genes had annotations that were associated with either defense or plant growth and development, or both. A total of 16 genes showed significant differences in transcript abundance about 2-fold, where 13 genes exhibited increased transcript abundance and 3 genes showed a decrease in transcript abundance (Table 2).

**Table 2:** Genes exhibiting nearly 2-fold changes in transcript abundance in *ABR17 *transgenic *A. thaliana *seedlings

AGI^*a*^	Operon annotation	log 2 ratio	SE^b^	p Value
At5g20230	ATBCB (Arabidopsis blue-copper-binding protein)	1.55	0.14	1.57E-03
At4g36060	BHLH family protein	1.49	0.19	4.33E-03
At5g44420	PDF1.2 (Plant defensin 1.2)	1.4	0.38	1.48E-02
At5g42040	Putative 26S proteasome non-ATPase regulatory subunit	1.38	0.41	2.04E-02
At4g22450	Unknown protein	1.37	0.17	3.96E-03
At3g45970	ATEXLA1 (A. thaliana expansin like A1)	1.32	0.16	1.08E-03
At5g01920	STN8 (State transition 8); KINASE	1.24	0.22	4.47E-03
At2g26010	PDF1.3 (Plant defensin 1.3)	1.17	0.35	1.97E-02
At5g10040	Unknown protein	1.04	0.31	2.79E-02
At1g75830	PDF1.1 (Plant defensin 1.1)	1.04	0.3	1.72E-02
At2g26020	PDF1.2B (Plant defensin 1.2B)	0.96	0.26	1.47E-02
At1g07135	Glycine rich protein	0.95	0.19	7.89E-03
At1g01560	Mitogen-activated protein kinase (MPK11), putative	0.94	0.1	1.08E-02
At5g48850	Male sterility MS5 family protein	-0.99	0.17	9.96E-03
At1g56430	Puatative, nicotianamine synthase	-1.13	0.08	8.78E-04
At3g56980	ORG3 (OBP3-resposnive gene 3)	-1.36	0.13	1.91E-03

All expression ratios are significant (α = 0.05) and are in a log2 scale where fold change is *ABR17*/WT.

AGI^*a *^– *Arabidopsis *Genome Initiative SE^b^- Standard error

Among the highly induced transcripts in transgenic seedlings that were putatively related to defense responses (Table 2), we detected 5 members of the plant defensin (PDF) family which exhibited an increased in abundance ~2–3-fold in the transgenic line. PDFs are small (45–54 amino acids), highly basic cysteine-rich peptides belonging to the large defensin family, and are present throughout the plant kingdom. These proteins are known for their involvement in ancestral non-specific innate immune defense system [32]. In addition to being involved in mediating plant responses to pathogens, defensins may also play an important role in plant growth and development. For example, the constitutive expression of *AtPep1 *induced the expression of *PDF1.2 *which resulted in better root development in *A. thaliana *suggesting that plant defensins may regulate root development [32].

Another interesting transcript that exhibited increased abundance (2-fold; Table 2) in *ABR17*-transgenic plants was a putative mitogen-activated protein kinase (*MAPK*). MAPK cascades are known to play crucial roles in physiological processes such as cell growth, cell cycle regulation and developmental control as well as plant defense signaling [33]. They are also known to activate WRKY type transcription factors that are involved in transcriptional activation of disease resistance genes [34]. Indeed, we have observed a modest, but elevated expression of four genes belonging to the *WRKY *family and disease resistance protein (Additional file 1).

We also observed increased transcript abundance for several genes involved in plant growth and development (Table 2). For example, expansins were detected as highly induced transcripts in *ABR17*-transgenic *A. thaliana *(Table 2). Expansins are cell wall proteins that are known to induce pH-dependent plant cell wall extension and stress relaxation [35]. The expansins have been related to cell differentiation in tissues such as xylem, leaf primordia and root hairs [36-38]. Previous studies on transgenic plants expressing expansin genes have demonstrated precocious leaf development, longer petioles and larger leaf blades [39,40].

Glycine-rich proteins (*GRP*s) were also detected among growth related genes that whose transcripts increased in abundance in *ABR17*-transgenic plants (Table 2). GRPs consist of quasi-repetitive glycine-rich domains, most commonly GGGX, GGXXXGG or GXGX repeats [41]. Some GRPs have been reported as structural components of the plant cell walls based on their co-localization with cell wall [42]. GRPs have also been reported to be activated by osmotic stress [43], cold shock [44] and wounding [45].

The genes that exhibited significant enhanced expression in *ABR17*-transgenic plants also included genes for proline-rich protein (*PRP*) family, xyloglucon endotransglycosylase (*XTH*), glycosyl hydrolase (GH), phytosulfokine precursor 2 (*PSK2*), No Apical Meristem (*NAM*) protein family and glutaredoxins (Additional file 1). PRPs represent a family of structural cell wall proteins that have been implicated in various plant developmental processes [46,47]. Similarly, XTH and GH family genes are involved in structuring xyloglucan cross-links in plant cell wall and plant development [[37,48] and [49]]. The *PSK2 *gene is also involved in cell growth and differentiation [50-52]. Similarly, the *NAM *gene product is required for shoot apical meristem (SAM) formation during embryogenesis as well as for normal flower development [53-55]. Glutaredoxins have also been demonstrated to be involved in flower development, probably by mediating post-translational modifications of target proteins required for normal petal organ initiation and morphogenesis [56]. Our current observations that the significantly (albeit modest) higher expression of the above mentioned genes related to growth and development including flowering correlates well with the observed phenotypes which include early flowering, increased lateral branching and seed pods as observed in *ABR17*-transgenic *A. thaliana *(Figure 1C).

### A role for cytokinins in ABR17-induced changes in gene expression?

Interestingly, members of most of the gene families described above (defensin, expansin, *MAPK*, *NAM*, *WRKY*, *GRP*, *PSK2 *and Glutaredoxin) that are involved in plant defense as well as growth and development, have been previously reported to be regulated by CKs. For example, genome-wide expression profiling of immediate-early and delayed CK- response genes of *A. thaliana*, has led to the identification of many genes that are up- regulated by CKs including members of expansin (*At1g69530*), *GRP*s (*At2g21060*), *NAM *(*At4g27410*), F-box protein (*At3g61060*), *ERBF*s, putative ring zinc finger protein (*At1g76410*), a member of the *bHLH *family (*At2g18300*), blue copper binding protein (*At5g20230*) and *PSK2 *[57]. The blue copper binding protein (*At5g20230*) and putative ring zinc finger protein (*At1g76410*) identified by Brenner *et al*. (2005) [57] as CK-induced were observed to be up-regulated in our microarrays analysis. Similarly, gene expression analysis of transgenic *A. thaliana *seedlings transformed with bacterial isopentenyl transferase (*IPT*) [58] gene revealed increased transcript abundance for many members of *MAPK *and *WRKY *gene families, which included the specific *WRKY *gene – *At1g80840 *that has been detected in our microarray studies as being induced by *ABR17 *expression (Additional file 1). Another investigation into CK action in *Arabidopsis *has demonstrated increased expression of genes for cytochrome P450, *PDF*, expansin, patatin, *WRKY *members and putative disease resistance protein in response to CKs [59]. Therefore, it is apparent that several genes whose transcript levels were modulated by *ABR17 *expression in *A. thaliana *have been previously reported in the literature as being CK-responsive, there by suggesting an important role for CK-mediated gene expression in ABR17 action *in planta*.

### Second set of transcriptional profiling: genes responsive to salt stress in WT *A. thaliana*

Microarray- based analyses of the salt responses in *Arabidopsis *have been published in several reports. However, most of these studies have investigated responses to very short-term exposure to salt. In this study, we report the transcriptional changes in *A. thaliana *as a result of long-term, continuous exposure to 100 mM NaCl. Here, we allowed *A. thaliana *seeds to germinate and grow on semi-solid medium in the presence of 100 mM NaCl for 2 weeks, and the RNA extracted from whole seedlings were used for cDNA synthesis and subsequent microarray analysis. The results from microarray analysis of salt treated WT *Arabidopsis *seedlings (Additional file 2) agreed with previous studies using similar approaches [60,61]. We identified 163 genes that showed more than four fold changes in transcript abundance which have been previously reported as being responsive to salt. Our results, therefore, indicate that both short-term "shock" treatments with NaCl as well as long-term treatment used in this study elicit similar responses in *A. thaliana *at the transcript level (Additional file 2).

Members of protease inhibitor/lipid transfer protein (LTP) family were seen among highly up regulated and/or down regulated genes. At least five members showed increase in transcript abundance and 1 member showed decrease in transcript abundance of more than 4 fold (Additional file 2). LTP genes contain ABA-responsive (ABRE) element (GTACGTGG) and are induced by abscisic acid (ABA) [62,63]. It has been reported in the literature that NaCl, mannitol or ABA treatments induce the expression of a gene encoding an LTP-like protein in tomato [62,64]. In addition, the changes in the expression of LTP genes during salt stress have been previously reported [60,61]. Although, most of the LTP genes were up regulated after short term treatment with salt, they were found to be down regulated after 24 h of salt treatment [60,61]. From our studies, it appears that many of the LTP genes will get up regulated in response to long term stress as a result of expected increase in ABA levels.

Other major groups of genes with increase in transcript abundance following NaCl treatment included two members of glycosyltransferases (GTs) and five members of glycoside hydrolases (GHs). GTs and GHs are major families of carbohydrate-active that play a primary role in structuring xyloglucan cross-links in plant cell wall [48,49]. They have been previously reported to be induced by salinity stress in plants and this has been implicated in drought and salt tolerance in *A. thaliana *[49,60]. Other genes exhibiting increased transcript abundance included ribonuclease RNS1, osmotin-like protein, hydroxycinnamoyl benzoyltransferase-related, oxidoreductase, 2OG-Fe(II) oxygenase family, glutathione transferase and zinc finger (C3HC4-type RING finger) protein family (Additional file 2). Similarly, the genes which showed decrease in transcript abundance more than 4 fold included many photosynthetic genes, plant defensins, heat shock proteins, auxin-induced proteins, disease resistance protein, Bet v I allergen family and bHLH protein. These results are once again consistent with the previously reported results from microarray-based investigation into salinity stress responses [60,61].

### Third set of transcriptional profiling: genes responsive to salt stress in presence of ABR17

The results from microarray analysis of salt treated *ABR17 *transgenic *A. thaliana *seedlings are presented in Tables 3 and 4. We identified 129 genes showing either increase or decrease in transcript abundance more than 4-fold, which included transcription factors (15), stress responsive genes (16), carbohydrate and cell wall metabolism (8), electron transport and oxidoreductases (6), lipid metabolism (3), protein and amino acid metabolism (9), proteins involved in transport across membranes (12) and 60 unknown or unclassified genes.

**Table 3:** Genes exhibiting > 4-fold change in transcript abundance in 100 mM NaCl treated *ABR17 *transgenic seedlings

AGI^*a*^	Operon annotation	Gene mean	SE^*b*^	p Value
**Transcription factors**				
At5g43650	bHLH protein family	4.67	0.51	1.22E-02
At1g43160	AP2 domain transcription factor RAP2.6	4.52	0.5	1.22E-02
At3g15500	ATNAC3 (Arabidopsis NAC domain containing protein 55)	3.8	0.28	8.39E-04
At1g10585	Transcription factor	3.32	0.2	4.55E-04
At3g43180	zinc finger (C3HC4-type RING finger) protein family	3.06	0.6	7.05E-03
At1g21910	AP2 domain-containing transcription factor family protein	3.01	0.04	2.66E-07
At1g52890	ANAC019 (Arabidopsis NAC domain containing protein 19)	2.96	0.46	7.40E-03
At5g13330	RAP2.6L (Related to AP2 6L)	2.86	0.11	1.33E-05
At4g05100	ATMYB74 (MYB domain protein 74)	2.53	0.29	3.21E-03
At2g38340	AP2 domain transcription factor, putative (DREB2)	2.4	0.27	3.08E-03
At2g46680	ATHB-7 (*Arabidopsis thaliana *HOMEOBOX 7)	2.16	0.02	9.75E-07
At2g38470	WRKY family transcription factor	2.05	0.21	1.75E-04
At4g17460	HAT1 (HOMEOBOX-leucine zipper protein 1)	-2.15	0.26	3.96E-04
At2g33810	SPL3 (SQUAMOSA PROMOTER binding protein-like3)	-2.22	0.54	2.55E-02
At1g62360	STM (shoot meristemless)	-2.76	0.35	1.44E-03

**Stress response**				

At2g03760	Steroid sulfotransferase	3.71	0.1	3.35E-06
At5g43570	Serine protease inhibitor family protein	3.57	0.09	2.85E-06
At4g04220	Disease resistance family protein	3.43	0.16	2.40E-04
At4g37990	Mannitol dehydrogenase (ELI3-2), putative	2.85	0.48	1.96E-03
At4g11650	Osmotin-like protein (OSM34)	2.36	0.17	3.95E-05
At5g39580	Peroxidase, putative	2.32	0.32	5.27E-03
At2g33380	RD20 (Responsive to dessication 20)	2.22	0.27	4.30E-04
At5g59820	Zinc finger protein	2.19	0.52	8.55E-03
At2g02990	Ribonuclease, RNS1	2.13	0.1	2.94E-05
At1g08830	Copper/zinc superoxidase dismutase (CSD1)	2.09	0.18	8.11E-05
At5g42180	Peroxidase, putative	-2.22	0.54	1.45E-02
At4g18780	CESA8 (Cellulase synthase 8)	-2.34	0.1	1.52E-04
At3g22231	PCC1 (Pathogen and circadian controlled 1)	-2.5	0.42	1.95E-03
At2g11810	MGDG synthase (MGD3), putative	-2.66	0.1	1.12E-04
At1g23130	Bet v I allergen family	-3.48	0.16	4.19E-06
At4g14400	ACD6 (Accelerated cell death 6)	-4.33	0.97	2.12E-02

**Carbohydrate and cell wall metabolism**				

At4g25810	Xyloglucan endotransglycosylase (XTR-6)	4.68	0.19	1.53E-04
At3g60140	Glycosyl hydrolase family 1 protein	4.19	0.1	3.46E-05
At2g36780	UDP-glycosyltransferase family	2.87	0.12	1.75E-04
At2g43620	Glycosyl hydrolase family 19 (chitinase)	2.81	0.4	9.08E-04
At4g16260	Glycosyl hydrolase family 17	2.49	0.11	2.06E-04
At4g26530	Fructose-bisphosphate aldolase, putative	-2	0.14	3.06E-05
At4g02290	Glycosyl hydrolase family 9	-2.22	0.25	3.11E-04

**Electron transport & Oxidoreductase**				

At2g37770	Aldo/keto reductase family	3.05	0.11	1.02E-05
At1g30700	FAD-linked oxidoreductase family	2.58	0.21	1.14E-03
At5g05600	Oxidoreductase, 2OG-Fe(II) oxygenase family	2.42	0.33	5.06E-03
At1g17020	SRG1 (Senescence-related gene 1)	2.26	0.08	1.35E-06
At2g45570	Cytochrome p450 family	2.11	0.34	8.70E-03
At5g20230	Arabidopsis blue-copper-binding protein	2.1	0.11	6.36E-06

**Lipid metabolism**				

At5g14180	Lipase family protein	2.78	0.45	1.59E-03
At1g54010	Myrosinase-associated protein, putative	2.24	0.69	4.77E-02
At3g02040	SRG3 (Senescense related gene 3)	-2.66	0.16	1.24E-05

**Protein and amino acid metabolism**				

At3g25250	Protein kinase family	2.54	0.38	6.62E-03
At4g04490	Protein kinase family protein	2.51	0.79	4.96E-02
At4g08870	Arginase – related	2.45	0.1	2.31E-06
At1g26970	Protein kinase, putative	2.39	0.09	1.50E-06
At1g76600	Similar to unknown protein (*Arabidopsis thaliana*)	2	0.58	2.59E-02
At1g21270	Protein serine/threonine kinase	-2.06	0.24	3.37E-04
At1g65800	ARK2 ((Arabidopsis receptor kinase 2)	-2.33	0.17	1.73E-04
At4g10540	Subtilase family protein	-2.36	0.07	5.74E-06
At4g21640	Subtilase family protein	-2.45	0.34	2.04E-03
At4g21650	Subtilase family protein	-2.49	0.65	3.13E-02

**Transport**				

At2g38530	Protease inhibitor/lipid transfer protein (LTP) family	3.91	0.16	2.35E-06
At4g12500	Protease inhibitor/lipid transfer protein (LTP) family	3.34	0.28	7.67E-05
At4g12490	Protease inhibitor/lipid transfer protein (LTP) family	3.32	0.29	9.06E-05
At3g50930	AAA-type ATPase family	3	0.19	1.86E-05
At4g12470	Protease inhibitor/lipid transfer protein (LTP) family	2.8	0.29	1.88E-04
At2g04070	MATE efflux protein family	2.67	0.31	3.24E-03
At5g43610	ATSUC6 (Sucrose-proton symporter 6)	2.5	0.33	6.37E-04
At3g51860	Cation exchanger, putative (CAX3)	2.2	0.24	2.64E-03
At2g04080	MATE efflux protein – related	2.1	0.32	1.17E-03
At4g12480	Protease inhibitor/lipid transfer protein (LTP) family	2.09	0.23	2.45E-04
At4g21680	Peptide transporter – like protein	2.03	0.76	4.41E-02
At5g19530	Spermine synthase (ACL5)	-2.02	0.14	2.72E-05

All expression ratios are significant (α = 0.05) and are in a log2 scale where fold change is salt treated *ABR17*/control *ABR17*. AGI^*a *^– *Arabidopsis *Genome Initiative SE^*b *^– Standard error

**Table 4:** Unknown/unclassified genes exhibiting > 4-fold changes in transcript abundance in NaCl-treated *ABR17 *transgenic seedlings

AGI^*a*^	Operon annotation	Gene mean	SE^*b*^	p value
At3g02480	ABA-responsive protein-related [Arabidopsis thaliana]	4.55	0.5	7.93E-04
At2g34600	Unknown protein	4.25	0.66	2.30E-02
At5g24640	Unknown protein	4.15	0.28	2.41E-05
At5g43580	Serine-type endopeptidase inhibitor put in unknown	3.73	0.64	2.14E-03
At4g13220	Similar to OS12G0276100	3.7	0.29	2.26E-04
At4g33720	Pathogenesis-related protein, putative	3.54	0.36	1.96E-04
At3g13600	Calmodulin-binding family protein	3.32	0.69	1.73E-02
At4g39670	Similar to ACD11 (Accelerated cell death 11)	3.2	0.12	1.02E-05
A023734_01	Putative ubiquitin-conjugating enzyme	2.63	0.52	1.47E-02
At5g38940	Germin-like protein, putative	2.59	0.34	4.76E-03
At1g66400	Calmodulin-related protein, putative	2.58	0.15	5.88E-05
At1g73260	Trypsin inhibitor -related	2.57	0.37	2.19E-03
At2g36770	Glycosyltransferase family	2.54	0.17	6.14E-04
At5g01920	STN8 (State transition 8)	2.52	0.07	4.94E-05
At4g01430	Nodulin MtN21 family protein	2.5	0.19	9.70E-04
At3g28210	Zinc finger protein (PMZ) -related	2.42	0.27	8.23E-04
At2g32200	Similar to unknown protein (Arabidopsis thaliana)	2.34	0.12	6.21E-06
At1g35140	Phosphate-induced (phi-1) protein -related	2.33	0.44	3.32E-03
At1g23710	Similar to unknown protein (Arabidopsis thaliana)	2.31	0.15	1.19E-04
At5g42830	Hydroxycinnamoyl benzoyltransferase-related	2.3	0.12	3.05E-04
At1g53470	Mechanosensitive ion channel domain-containing protein	2.19	0.15	1.38E-04
At2g36800	Glucosyl transferase -related	2.18	0.16	4.37E-05
At4g24380	Hydrolase, acting on ester bonds	2.16	0.35	1.68E-03
At2g41640	Similar to unknown protein (Arabidopsis thaliana)	2.15	0.15	7.48E-04
At2g30840	2-oxoglutarate-dependent dioxygenase, putative	2.14	0.16	3.57E-05
At5g35510	Unknown protein	2.09	0.16	2.00E-04
At1g17380	Similar to unknown protein (Arabidopsis thaliana)	2.06	0.14	2.27E-05
At5g03210	Unknown protein	2.06	0.55	1.37E-02
At2g36790	Glucosyl transferase -related	2.04	0.49	1.44E-02
At3g03820	Auxin-induced protein, putative	-2	0.24	1.08E-03
At1g12080	Similar to unknown protein (Arabidopsis thaliana)	-2.04	0.33	1.60E-03
At1g78020	Senescence-associated protein -related	-2.07	0.11	9.14E-06
At2g32870	MEPRIN and TRAF homology domain-containing protein	-2.12	0.31	2.40E-03
At5g22580	Expressed protein	-2.16	0.22	1.74E-04
At5g64770	Similar to 80C09_10 (*Brassica rapa*)	-2.19	0.19	8.40E-05
At2g14560	Similar to unknown protein (Arabidopsis thaliana)	-2.22	0.3	6.53E-04
At4g00755	F-box protein family	-2.27	0.14	1.40E-05
At3g32130	Similar to unknown protein (Arabidopsis thaliana)	-2.3	0.17	1.70E-04
At3g45160	Unknown protein	-2.33	0.17	3.44E-05
A003747_01	Histone H2B, putative	-2.36	0.18	1.89E-04
At4g39800	Myo-inositol-1-phosphate synthase	-2.48	0.12	5.20E-06
At2g41090	Calmodulin-like calcium binding protein (CaBP-22)	-2.48	0.14	1.03E-05
At3g04210	Disease resistance protein (TIR-NBS class), putative	-2.55	0.09	1.04E-06
At5g18030	Auxin-induced (indole-3-acetic acid induced) protein, putative	-2.58	0.24	1.15E-04
At5g42530	Similar to ECS1 (Arabidopsis thaliana)	-2.59	0.11	2.47E-06
At2g40610	ATEXPA8 (Arabidopsis thaliana expansin8)	-2.61	0.1	1.50E-05
At5g18080	Auxin-induced (indole-3-acetic acid induced) protein, putative	-2.61	0.26	1.68E-04
At1g67870	Glycine-rich protein	-2.64	0.1	1.56E-06
At1g29460	Auxin-induced (indole-3-acetic acid induced) protein, putative	-2.73	0.3	2.72E-04
At1g14880	Similar to unknown protein (Arabidopsis thaliana)	-2.79	0.63	1.14E-02
At1g29430	Auxin-induced (indole-3-acetic acid induced) protein family	-2.8	0.83	4.29E-02
At1g29510	Auxin-induced (indole-3-acetic acid induced) protein, putative	-2.88	0.19	2.18E-05
At2g25510	Unknown protein	-2.91	0.14	2.95E-05
At5g61980	ARF GTPase-activating domain-containing protein	-3.03	0.54	5.01E-03
At2g04460	Retroelement pol polyprotein -related	-3.15	0.28	1.54E-03
At1g67860	Similar to unknown protein (Arabidopsis thaliana)	-3.16	0.25	5.21E-05
At5g18010	Auxin-induced (indole-3-acetic acid induced) protein, putative	-3.21	0.12	1.39E-06
At5g18020	Auxin-induced (indole-3-acetic acid induced) protein, putative	-3.26	0.2	1.46E-05
At5g35480	Unknown protein	-3.76	0.37	5.27E-04
At4g14400	ACD6 (Accelerated cell death 6)	-4.32	0.97	2.12E-02

All expression ratios are significant (α = 0.05) and are in a log2 scale where fold change is salt treated *ABR17*/control *ABR17*.

AGI^*a *^– *Arabidopsis *Genome Initiative SE^*b *^– Standard error

Transcriptional factors are necessary for the proper transcriptional regulation in response environmental cues [65] and those exhibiting significant increases in transcript abundance included bHLH, 4 members of AP2 related, 2 members of NAM, zinc finger (C3HC4-type RING finger) protein family, ATMYB74 (MYB domain protein 74), ATHB-7 (*Arabidopsis thaliana *HOMEOBOX 7), and WRKY families. *bHLH092 *has been indicated among the highly induced transcripts in response to NaCl treatment in the previous transcriptomic studies and are suggested to be important regulators of the NaCl-stress response in *Arabidopsis *[60]. The APETALA2 (AP2) domain defines a large family of transcription factors which play important roles in plant growth, development as well as stress tolerance [66-71].

Similarly, as previously stated, NAM genes have been found to be induced by abiotic stresses implying roles in stress responses in addition to those in plant growth and development [72,73]. NAM/NAC proteins belong contain highly conserved NAC (for NAM, ATAF1, 2, and CUC2) domains in their N-terminal regions, that specifically binds target DNA [54]. It has also been demonstrated that NAC transcription factors are ABA-responsive [74,75] and are also induced by other plant hormones like NAA and ethylene [75-78]. Overexpression of NAC genes has been shown to result in an increase in lateral roots, and tolerance to abiotic stresses like drought and salt stress. NAC genes are believed to exert their stress ameliorating activity through the regulation of stress-inducible genes [77-79]. Similarly, the WRKY family TF genes and myb family genes are known to be biotic and/or abiotic stress responsive [60,80]. Thus, it is possible that the increased tolerance of *ABR17*-transgenic seedlings to NaCl is the combined effect of the modulation of the levels of abundance of transcripts for these transcription factors with demonstrated roles in stress tolerance.

The highest transcript abundance of any gene observed in salt treated *ABR17 *plants was *XTR-6*, which showed 4.7-fold increase, compared to the untreated *ABR17*-transgenic line. Xyloglucan endo-transglycosylase (XET) has been suggested to be a key enzyme involved in the modification of the xyloglucan cross-links that controls the strength and extensibility of the plant cell wall [81]. Three members of GH family were also seen among genes which up regulated more than 4-fold change (Table 3). The importance of GHs genes in plant stress [49,60] has already been discussed in the previous section.

Others salt responsive genes in the *ABR1*7-transgenic line included osmotin, mannitol dehydrogenase, steroid sulfotransferases and RD20 (Table 3) which are known to be regulated by ABA, are expressed in salt-stressed plants and have been used to engineer salinity tolerance [82-86]. In addition, we also observed increase in transcript abundance for ribonuclease- RNS1, peroxidases, copper/zinc superoxidase dismutase (CSD1), cytochrome p450 family, MATE efflux protein and protein kinases which have been previously demonstrated to accumulate in salt treated tissues by others [60,61]. From our microarray results, it appears that many genes involved in mediating responses to salinity stress are increased in transcript abundance as would be expected.

### Comparison of salt responses in WT and ABR17 transgenic seedlings

Although transcriptional changes were almost similar both in salt treated- *ABR17 *and WT seedlings, the transcript abundance of some genes exhibited significant differences in both the trend as well as the degree of modulation of transcript abundance (Table 5). For instance, as mentioned previously, transcript abundance of xyloglucan endotransglycosylase (*XTR-6*) (*At4g25810*) increased 4.7-fold in salt treated *ABR17 *seedlings, whereas it showed only a 2.4-fold increase in salt-treated WT seedlings (Table 5). Similarly, AP2 domain related transcription factor RAP2.6 (*At1g43160*) increased 4.5-fold in salt treated ABR17 compared to 1.67-fold in treated WT plants. The expressed proteins- ABA-responsive protein-related (*At3g02480*) and unknown protein (*At5g24640*) also showed increase in transcript abundance of at least 4-fold in salt-treated *ABR17 *transgenic line compared to the 2-fold increase observed for the WT in response to salt. Other genes which exhibited increase in transcript abundance of more than 2 fold in salt-treated *ABR17 *transgenic but showed less abundance in treated WT included pathogenesis-related protein, (*At4g33720*) and glutamine-dependent asparagine synthetase (*At3g47340*). On the other hand, the retroelement pol polyprotein (*At2g04460*) with unknown function showed a down regulation of more than 2-fold in salt-treated transgenic *ABR17 *line, but was up regulated in salt treated WT *Arabidopsis *plants.

**Table 5:** Comparison of changes in gene expression between NaCl-treated WT and *ABR17 *transgenic Arabidopsis seedlings.

AGI^*a*^	Operon annotation	*ABR17*	SE^*b*^	WT	SE^*b*^	*ABR17*-WT	p value
		log2 ratio		log2 ratio			

At4g25810	Xyloglucan endotransglycosylase (XTR-6)	4.68	0.39	2.35	1.31	2.33	6.47E-03
At3g02480	ABA-responsive protein-related	4.55	1.11	2.4	0.6	2.15	1.16E-02
At1g43160	AP2 domain transcription factor RAP2.6	4.52	0.87	1.67	0.6	2.85	1.55E-02
At5g24640	Unknown protein	4.15	0.28	2.58	0.36	1.57	7.00E-03
At4g33720	Pathogenesis-related protein, putative	3.54	0.89	1.19	0.77	2.35	8.68E-04
At3g47340	Glutamine-dependent asparagine synthetase	2.04	0.12	-0.02	0.45	2.06	8.68E-04
At2g29500	Small heat shock protein -related	1.69	0.93	-0.21	0.61	1.91	2.98E-03
At1g59860	Heat shock protein, putative	1.59	0.8	-1.59	1.03	3.17	2.10E-04
At5g12030	*A. thaliana *mRNA for 17.6kDa HSP protein	1.41	0.39	-0.7	0.81	2.11	1.82E-03
At5g51440	Heat shock protein, putative	1.32	0.6	0.08	0.3	1.24	2.78E-03
At3g09440	Heat shock protein hsc70-3 (hsc70.3)	1.22	0.24	-3.03	0.53	4.25	1.98E-06
At5g56010	Heat shock protein, putative	1.19	0.35	-1.06	0.36	2.26	1.57E-06
At2g26150	Heat shock transcription factor family	1.06	0.59	-0.55	0.69	1.62	1.73E-03
At1g74310	Heat shock protein 101 (HSP101)	1.06	0.27	-2.42	0.75	3.48	4.04E-05
At5g48570	Peptidylprolyl isomerase	1.02	0.3	-2.18	0.91	3.19	1.84E-04
At5g44420	Plant defensin protein, putative (PDF1.2a)	0.9	0.23	-2.51	0.51	3.41	5.74E-06
At5g44430	Plant defensin protein, putative (PDF1.2c)	0.8	0.25	-2.62	0.42	3.42	1.38E-07
At5g56030	Heat shock protein 81-2 (HSP81-2)	0.77	0.17	-1.52	0.34	2.29	1.46E-06
At2g26010	Plant defensin protein, putative (PDF1.3)	0.76	0.2	-2.95	0.38	3.7	1.38E-07
At3g12580	Heat shock protein hsp70	0.73	0.44	-1.93	1.1	2.66	1.55E-03
At5g56000	Heat shock protein 81.4 (hsp81.4)	0.64	0.2	-1.91	0.57	2.55	5.05E-05
At5g12020	Class II heat shock protein	0.61	0.29	-0.41	0.49	1.01	2.58E-03
At1g75830	Plant defensin protein, putative (PDF1.1)	0.58	0.16	-1.72	0.51	2.3	4.23E-05
At4g11660	Heat shock factor protein 7 (HSF7)	0.53	0.35	-0.77	0.75	1.3	6.99E-03
At5g02500	Heat shock protein hsc70-1 (hsp70-1)	0.52	0.16	-0.75	0.24	1.27	4.52E-06
At5g02490	Heat shock protein hsc70-2 (hsc70.2)	0.43	0.13	-1	0.27	1.43	7.67E-06
At2g19310	Small heat shock protein -related	0.39	0.22	-1.98	0.44	2.37	8.50E-06
At1g16030	Heat shock protein hsp70b	0.34	0.26	-0.77	0.22	1.11	1.42E-04
At2g04460	Retroelement pol polyprotein -related	-3.15	0.56	1.55	0.63	-4.7	5.32E-06

Footnote: All expression ratios are significant (α = 0.05) and in a log2 scale where fold change is salt treated *ABR17*/control *ABR17 *and salt treated WT/control WT.

*ABR17*- WT = Difference in log2 ratio of salt treated *ABR17*/control *ABR17 *and salt treated WT/control WT.

AGI^*a *^– *Arabidopsis *Genome Initiative SE^*b *^– Standard error

Interestingly, many members of heat shock protein (Hsp) family and PDF family showed the opposite response in salt-treated *ABR17*-transgenic seedlings compared to the salt-treated WT counterparts, with an increase of transcript abundance in salt-treated *ABR17*-transgenic (Table 5). This difference in the direction of the response in gene expression i.e. induction in the transgenic seedlings versus the repression in the WT may have important consequences with respect to the ability to tolerate salinity (and perhaps other) stress. For example, the Hsp family contains chaperones, which have important roles in protein folding, assembly and in the disposal of unwanted nonfunctional proteins. Hsps are usually induced by environmental stress, and the accumulation of Hsps coincides with enhanced stress tolerance [60,87-91]. In addition, transgenic *Arabidopsis *plants overexpressing *AtHSP17.7 *accumulate high levels of AtHSP17.7 protein and show enhanced tolerance to drought and salinity [89,90]. The abundance of Hsps in plants and their functional characteristics suggest that Hsps play an important role in plant stress tolerance. Thus, the increased abundance of HSP transcripts in the *ABR17*-transgenic seedlings may be important for the increased tolerance of this line to the imposed stress.

The up regulation of PDFs in *ABR17*-transgenic *A. thaliana *grown under normal conditions (Table 2) and their importance in growth and development has already been discussed earlier. The literature also supports a role for PDFs in stress tolerance [32,91]. Most of the previously characterized PDFs exhibit anti-fungal, antibacterial, anti- insect and protease inhibitor activity [92]. However, the halophyte salt cress (*Thellungiella halophila*), a relative of *Arabidopsis *over expresses PDFs under normal conditions and hence defensins are believed to play a role in salt tolerance [93]. It is therefore possible that the observed relatively tolerant phenotype of *ABR17*-transgenic seedlings could be due, at least in part, to the elevated expression of *XTR6*, *RAP2.6 *transcription factors, unknown proteins (*At3g02480, At5g24640*), Hsp and PDF gene(s). Additional studies are underway in our laboratory to precisely characterize the role(s) of the above-mentioned genes in stress tolerance of *ABR17*- transgenic *A. thaliana *seedlings.

### qRT-PCR validation of microarray observations

**Figure 3:**
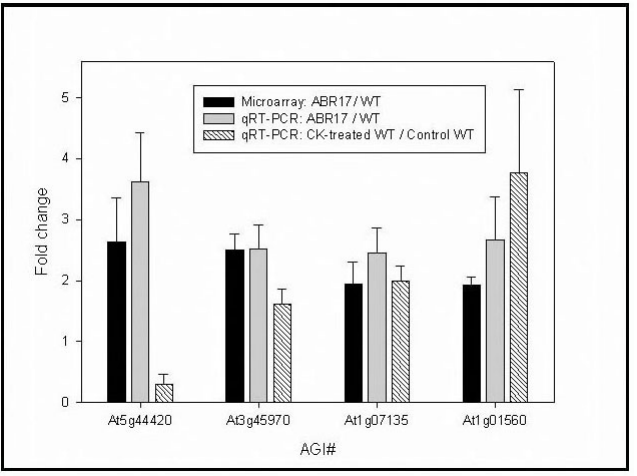
**Pea *ABR17*-modulated transcriptional changes of selected genes**. Transcriptional changes of a selected number of transcripts as identified by microarrays, and their validation using qRT- PCR and effects of CK on these genes in WT *A. thaliana *is given here. The values represented in the graph are fold changes of transcript abundance between transgenic *ABR17-A. thaliana *seedlings versus WT seedlings grown under normal conditions. Error bars are standard error of fold changes driven from (n = 3) three biological replicates. The AGI annotations are as follows: **At5g44420**-Plant defensin protein family member PDF1.2, Low-molecular-weight cysteine-rich (LCR77); **At3g45970**-ATEXLA1 (*A*. *thaliana *expansin-like A1); **At1g07135**-Glycine rich protein; and **At1g01560**-ATMPK11.

In order to confirm the fact that CK-responsive genes were indeed up-regulated in the *ABR17*-transgenic lines, we performed qRT-PCR experiments with the following genes: plant defensin protein (*PDF1.2a*, *At5g44420*), expansin (*EXPL1*, *At3g45970*), *GRP *(*At1g07135*) and putative *MAPK 11 *(*Atg01560*) using qRT-PCR. Among the CK- inducible genes identified from our first set of microarray experiments we chose the above 4 genes for qRT-PCR as their transcripts were observed to be at least ~2-fold (1 in log2 ratio) or higher in the transgenic line compared to WT. Our microarray analysis revealed increase in transcripts for defensin, expansin, *GRP *and MAPK in pea *ABR17 *seedlings by 2.6, 2.5, 1.9 and 1.9-fold, respectively (Figure 3). Our qRT-PCR results were consistent with the microarray data and showed up-regulation of defensin, expansin, *GRP *and *MAPK *by 3.6, 2.5, 2.5 and 2.7-folds, respectively (Figure 3). From these results it is apparent that all the four genes that were up-regulated in our microarray analysis also demonstrated up- regulation in the qRT-PCR relative expression experiments (Figure 3).

Because of the fact that the specific members of gene families whose transcripts were detected to be modulated by *ABR17 *cDNA expression in *A. thaliana *were not exactly identical to those specific members of these families identified by other studies, we wanted to investigate whether those specific members detected in our studies were indeed CK-inducible/repressible. In these experiments, we used WT *A. thaliana *tissue germinated and grown for 14-days medium supplemented with 5 μM zeatin for additional qRT-PCR experiments. This concentration of zeatin was chosen based on our earlier observations that it induced the largest phenotypic responses in *A. thaliana *when exogenously applied [8]. It must also be noted that even though 5 μM zeatin was used in our experiments, it is difficult to estimate how much of this exogenously supplied CK actually gets into the seed in order to exert a physiological effect. From the results shown in Figure 3, it is apparent that the expression of *EXPL1 *(*At3g45970*), putative *MAPK *(*Atg01560*) and *GRP *(*At1g07135*) was up-regulated in response to exogenous zeatin by 1.6, 3.8, and 2-fold, respectively (Figure 3). In contrast, the expression of defensin gene was observed to be down-regulated (0.3-fold) in response to the exogenous application of CK (Figure 3). The results for expansin, *MAPK *and *GRP *are consistent with our microarray and qRT-PCR results with respect to increased transcript abundance in *ABR17*-transgenic *A. thaliana *previously shown to possess higher endogenous CK concentrations [8]. However, in the case of defensin, even though our microarray and qRT-PCR experiments revealed that this gene was up-regulated in the *ABR17*-transgenic line (Figure 3), its expression was not induced by exogenous CK (Figure 3). The reason for this discrepancy is not immediately clear; however, this may be due to the concentration as well as type of CK used for our exogenous experiments. Furthermore, as indicated previously, the amount of the exogenously supplied CK entering the seed to exert physiological affect may also be different from the concentrations required to elicit induction of this gene.

**Figure 4:**
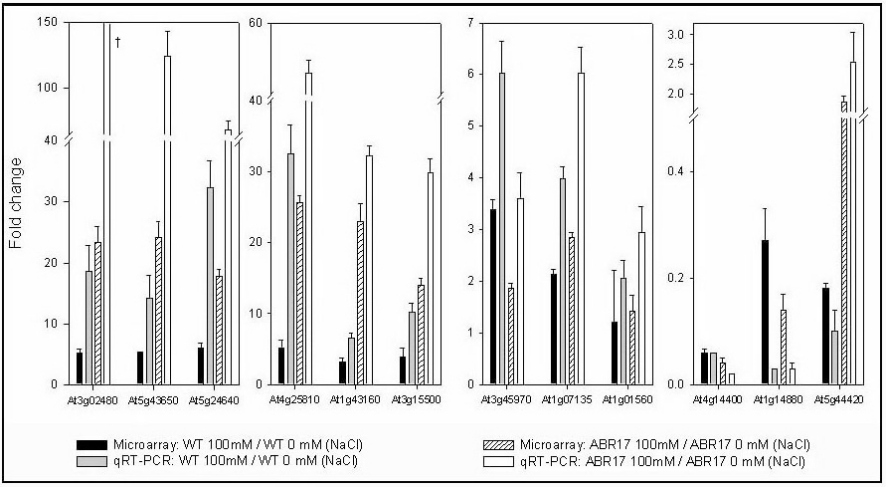
**Transcript abundance of selected genes in salt treated-WT and *ABR17 *transgenic *A. thaliana *seedlings**. The values represented in the graph are fold changes of transcript abundance as identified by microarrays and their validation using qRT-PCR, between salt treated (100 mM) seedlings versus untreated seedlings either in wild type or in *ABR17 *transgenic *A. thaliana*. Error bars are standard error of fold changes driven from (n = 3) three biological replicates. The AGI annotations are as follows:**At3g02480**-ABA-responsive protein-related; **At5g43650**-basic helix-loop-helix (bHLH) family protein; **At5g24640**-unknown protein; **At4g25810**-XTR6: Xyloglucan endotransglycosylase 6; **At1g43160**-ethylene-responsive transcription factor Related to Apetala 2.6 (Protein RAP2.6); **At3g15500**-ATNAC3 (*A. thaliana *NAC domain containing protein 55); **At4g14400**-ACD6 (Accelerated cell death 6); **At1g14880**-PLAC8 domain containing protein; **At5g44420**-Plant defensin protein family member PDF1.2; **At3g45970**- ATEXLA1 (*A. thaliana *expansin-like A1); **At1g07135**-Glycine rich protein; and **At1g01560**: ATMPK11 (*A. thaliana *MAP kinase 11). † : the fold change in here is 272.27 ± 58.5.

In order to confirm results from our second and third set of microarray analysis, we performed qRT-PCR experiments with the following 12 genes: unknown proteins (*At3g02480*; *At5g24640*; *At1g14880*), *XTR6 *(*At4g25810*), *bHLH *(*At5g43650*), *AP2 *domain transcription factor *RAP 2.6 *(*At1g43160*), *ATNAC3 *(*At3g15500*), *ACD6 *(*At4g14400*), *PDF1.2a *(*At5g44420*), *EXPL1 *(*At3g45970*), *GRP *(*At1g07135*) and *MAPK 11 *(*Atg01560*). The unknown proteins were chosen because expression of two of them (*At3g02480 *and *At5g24640*) were among highly induced transcripts in salt treated *ABR17*-trangenic line and also showed comparatively less but high level of transcript abundance in salt treated WT *A. thaliana *lines (Table 5, Figure 4). Two of them (*At1g14880 *and *At4g14400*) were among highly down regulated genes in both salt treated *ABR17*-transgenic line and WT *A. thaliana *lines (Table 3 and 4, Figure 4). Our qRT-PCR data showed similar trend as observed by microarrays for all the above-mentioned genes in both salt-treated *ABR17*-transgenic and salt-treated WT microarrays (Figure 4).

The genes *At3g02480, At5g24640, At1g14880 *and *At4g14400 *showed transcript abundance with fold changes of 5.26, 5.98, 0.27 and 0.06, respectively in our microarray analysis of salt treated WT *A. thaliana *(Figure 4). Our qRT-PCR analysis of salt treated WT *A. thaliana *showed transcript abundance of and 18.60, 32.42, 0.03 and 0.06-fold for genes *At3g02480, At5g24640, At1g14880 *and *At4g14400 *(Figure 4) compared to qRT-PCR indicated transcript abundance of 23.36, 17.80, 0.14 and 0.04-fold for genes *At3g02480, At5g24640, At1g14880 *and *At4g14400*, respectively (Figure 4). Our qRT-PCR analysis of salt treated *ABR17 A. thaliana *showed transcript abundance of 272.37, 67.49, 0.03 and 0.02-fold for genes *At3g02480, At5g24640, At1g14880 *and *At4g14400*, respectively (Figure 4). From these results, it is apparent that all the four genes showed the same trend both in our microarray analysis and qRT-PCR studies (Figure 4) although the absolute values were different with these two experimental methods.

The gene *XTR6 *(*At4g25810*) was selected because it was among one of the most highly induced transcripts of any gene on our salt treated *ABR17*-transgenic *A. thalina *microarray (Table 3, Figure 4). The genes *bHLH *(*At5g43650*), *RAP 2.6 *(*At1g43160*) and *ATNAC3 *(*At3g15500*) were chosen because their expression was the highest among any other transcription factors identified in response to salt in *ABR17*- transgenic line (Table 3, Figure 4). The genes *At4g25810*, *At5g43650*, *At1g43160 *and *At3g15500 *showed transcript abundance of 5.10, 5.29, 3.19 and 3.88-fold, respectively in microarray analysis of salt treated WT *A. thaliana*, while our qRT-PCR analysis of salt treated WT *A. thaliana *showed transcript abundance of 32.51, 14.17, 6.58 and 10.23- fold (Figure 4). Similarly, microarray analysis of salt treated *ABR17 A. thaliana *showed transcript abundance of 25.62, 24.17, 23.00 and 13.96 (Figure 4) and our qRT-PCR analysis values of 54.40, 124.30, 32.27 and 29.88- fold for genes *At4g25810*, *At5g43650*, *At1g43160 *and *At3g15500*, respectively (Figure 4). Our microarray analysis and qRT-PCR results showed the similar trend in both salt treated-*ABR17 *and WT samples (Figure 4), The genes *PDF1.2a, EXPL1*, *GRP*, and *MAPK 11 *were chosen as these were validated in our first set of microarrays (ABR17/WT under normal conditions). Once again, a similar trend was observed between microarrays and qRT-PCR analysis thus validating our microarray results.

### Relative expression of CK-biosynthetic genes (IPT and CKX) in ABR17-transgenic *A. thaliana*

As discussed earlier, our observations indicated that many of the genes identified in the transgenic plants as being up-regulated are from families that contain CK-responsive members. We have also previously reported higher endogenous concentrations of CK in this line [8], which suggested the possibility that this may be due to either enhanced *de novo *CK biosynthesis or decreased degradation. Specifically, the endogenous concentration of total CK in the transgenic line used in this study was ~1–3-fold higher, with the concentration of zeatin (cis and trans combined) being ~1.4-fold and IP being ~2-fold higher in these transgenic lines. However, we did not detect any *IPT *(involved in CK biosynthesis) or *CKX *(involved in CK degradation) genes as being significantly up- or down-regulated genes in our microarray experiments suggesting that the elevated endogenous CK concentrations previously reported may not be the result of increased or decreased activities of *IPT *and *CKX *genes, respectively. In order to confirm our microarray results and to lend additional support to our above- mentioned hypothesis with respect to the roles (or lack thereof) of *IPT *and *CKX *expression in *ABR17*-transgenic *A. thaliana*, we also performed qRT-PCR analysis of the expression of *IPT *and *CKX *genes using qRT-PCR. There are 9 known *IPT *genes and 7 known *CKX *genes but sequence of *CKX5 *and *7 *are very similar therefore we performed qRT-PCR analysis on the 9 *IPT *and 6 *CKX *genes. The results from these experiments are summarized in Table 6 and it is apparent that most of the *IPT *genes exhibit similar expression patterns in both transgenic and WT seedlings. The only exception appears to be *IPT 8 *where only 0.5-fold expression of this gene was observed in the transgenic line (Table 6). Similarly, *CKX *expression in the transgenic line was also quite similar to its expression in the WT (Table 6). Our results suggest that the differences in endogenous CK concentrations previously observed in the *ABR17*-transgenic line may not be the result of increased IPT or decreased CKX levels. However, frequently, there is no correlation between transcript abundance and protein levels and therefore it is possible that IPT and/or CKX protein concentrations may have been affected in the transgenic line resulting in increased endogenous CKs as a result of post-translational processes. However, our previously reported proteome studies on this transgenic line did not reveal any differences between transgenic and WT seedlings with respect to the levels of these proteins [19].

**Table 6:** *IPT *and *CKX *gene expression in *ABR17*-transgenic *A. thaliana*

Gene	Fold change *
*IPT1*	1.20 ± 0.28
*IPT2*	1.24 ± 0.17
*IPT3*	1.29 ± 0.17
*IPT4*	1.18 ± 0.47
*IPT5*	1.17 ± 0.26
*IPT6*	0.99 ± 0.32
*IPT7*	1.37 ± 0.37
*IPT8*	0.49 ± 0.09
*IPT9*	1.10 ± 0.19
*CKX1*	1.39 ± 0.30
*CKX2*	1.16 ± 0.31
*CKX3*	1.50 ± 0.44
*CKX4*	0.72 ± 0.18
*CKX5*	0.91 ± 0.22
*CKX6*	0.79 ± 0.11

*The expression of each gene in WT was normalized to 1 and fold change in transgenic line was calculated as described in Methods

It is possible that the activity of neither IPT nor CKX is responsible for the increased endogenous concentrations of CKs in the *ABR17*-transgenic lines and the increased endogenous CKs previously reported in the *ABR17*-transgenic lines may be the result of tRNA degradation by the previously demonstrated RNase activity of pea ABR17 protein [8]. Thus, an increase in free cellular CK would not necessarily involve enhanced IPT or reduced CKX activity; rather it may reflect an increased access to existing, yet tRNA-bound, CK.

## Conclusion

We have demonstrated that pea *ABR17 *cDNA expression modulates the level of a number of transcripts related to plant defense, growth and development, which may explain the observed phenotypic differences between WT and *ABR17*-transgenic *A. thaliana*. The gene expression of many transcription factors and defense responsive genes like Hsps and PDFs showed different degree and kind of response between salt treated-*ABR17 *transgenic and WT *A. thaliana*, which explains the observed enhanced germination and early seedling vigor in *ABR17 *transgenic lines, compared to its WT counterpart. Many of the genes exhibiting a 2-fold or higher increase in transcript abundance are known CK-responsive genes providing additional evidence of a role for CKs in ABR17 function. Furthermore, a detailed expression analysis of *IPT*s and *CKX*s revealed that the levels of these transcripts were similar in both WT and transgenic seedlings, suggesting the possibility that ABR17 modulates endogenous CKs through an, as of yet, uncharacterized mechanism including the possible degradation of tRNAs which contain CK moieties [94]. These possibilities are currently being investigated in our laboratory.

## Methods

### Plant material and growth conditions

Transformation of *A. thaliana *with the pea *ABR17 *cDNA and the generation of homozygous *ABR17*-transgenic *A. thaliana *(line 6.9) have been previously described [19]. This line (6.9) was one of the three independently derived transgenic lines that were characterized in that earlier study. The WT (ecotype WS) and transgenic *A. thaliana *plants were grown in the green house for observations as previously described [8]. Lateral branches were counted on plants from three independent biological replicates with at least 72 plants per replicate. Average number of days required for the opening of the first flower was also recorded on plants from three biological replicates with 36 plants in each replicate.

In order to measure root lengths of seedlings seeds of *A. thaliana *(line 6.9) and the WT were surface sterilized [8] and placed on half strength Murashige & Skoog (MS) medium [22] with or without salt (75 mM NaCl or 100 mM NaCl) in square dishes with grids. These dishes were placed vertically in a growth chamber (at 21°C and with light intensity of 250 μmol m^-2 ^s^-1^) and root lengths were measured after 10 days. The seeds of *ABR17 *and WT seeds were also grown on half strength MS medium with 0 or 100 mM NaCl to determine their fresh weight and chlorophyll and carotenoid contents in order to assess their ability to grow in the presence of salt. The length of the primary roots of 10-day-old seedlings from three independent biological replicates with at least sixty seedlings per replicate, were calculated using the Image J software (Image J, NIH, MD, USA).

Chlorophyll and carotenoids were extracted from the pooled 2-week-old tissue grown on MS media, using the procedure as described by Srivastava *et al*., 2006 [7]. Total chlorophyll was estimated using a nomogram [95] and total carotenoid was measured using the formula:

Δ*A*CAR_480 _= Δ*A*_480_+0.114Δ*A*_663_-0.638Δ*A*_645_

where *A *is the absorbance and CAR is the carotenoid content [96]. The fresh weight, chlorophyll and carotenoid were calculated using pooled tissue from three independent biological replicates. Percent germination after one week for *ABR17*-transgenic and WT seeds in the dark and in the presence of light (fluorescent light, 30 μmol m^-2 ^s^-1^) were compared in Petri dishes at RT. This experiment included three independent biological replicates with at least 45 seeds per replicate. All statistical analyses were performed using the Student's *t*-test procedure in SAS version 8e (Statistical Analysis System 1985).

Tissue for microarray analysis was obtained by placing surface sterilized seeds of *A. thaliana *(line 6.9) and the WT on half strength MS medium in Petri dishes with or without 100 mM NaCl at RT (21 ± 2°C) under continuous fluorescent light 30 μmol m^-2 ^s^-1 ^for 14 days. Seedlings (14-day-old) from three independently grown biological replicates in all three set of experiments (comparison of *ABR17*-transgenic with WT without any stress; comparison of salt treated WT with untreated WT; comparison of salt treated *ABR17 *transgenic with untreated transgenic) were removed from the MS plates, flash frozen in liquid nitrogen and stored at -80°C until used for RNA extraction.

### RNA extraction, cDNA synthesis and microarray analysis

In order to investigate the ABR17-induced gene expression changes under normal and salinity stress conditions, we conducted microarray analysis in three separate hybridization experiments. The first set (set I), consisted of comparison of cDNA samples prepared from *ABR17*-transgenic and WT seedlings, which were grown in the absence of any stress. Set II consisted of cDNA obtained from salt-treated samples of WT and untreated WT; and set III, cDNA samples of salt treated *ABR17*- and untreated *ABR17*-transgenic seedlings for hybridization to the oligo-nucleotide arrays. Each microarray experiment consisted of six hybridizations according to the principles of dye-swap design [97] on tissues across three biological replicates of the experiments.

RNA was isolated using the QIAGEN RNeasy Plant Mini Kit (Qiagen Inc., Mississauga, ON, Canada) from 2-week-old WT and *ABR17 *seedling tissue from all three set of experiments and the integrity of all RNA samples assessed by agarose gel (1.2%) electrophoresis. For microarray hybridization, 6 μg of total RNA was used to synthesize cDNAs using SuperScript^® ^II RT (Invitrogen Inc., Burlington, ON, Canada) with RT polyA-capture primers in 3D Array 900TM (Genisphere Inc., Hatfield, PA, USA). In these microarray experiments, 70-mer oligonucleotide arrays were used which contained 26,090 probes (Array-Ready Oligo Set for Arabidopsis genome Version 1.0, Qiagen Operon, Alameda, CA, USA), plus additional probes for quality control. Oligonucleotide arrays were spotted on superamine aminosilane-coated slides (TeleChem International Inc., Sunnyvale, CA, USA). Each pair of samples within each of the three biological replicates was labeled in a reciprocal dye-swap design, for a total of 18 hybridizations (overnight, at 52°C) in all three sets of experiments. Slides were scanned using ArrayWoRx^e ^(Applied Precision, Issaquah, WA, USA) and spot intensities were measured, quantified, normalized and analyzed using TM4 [98]. Spots with intensity ratios that differed significantly from 0 (log2 scale) were identified by Student's *t*-test. This procedure highlights the spots that demonstrated statistically significant differential expression between the different samples. The raw microarray data of 18 hybridizations as well as the protocols used to produce the data were deposited in the ArrayExpress database [ArrayExpress: E-MEXP1024 and E-MEXP1566].

### Quantitative real-time PCR (qRT-PCR) validation of microarray data

Primers for qRT-PCR were designed using the Primer Express software (Applied Biosystems Inc., Foster City, CA, USA) to ensure that PCR products of approximately 70–80 bp were generated (Additional file 3). cDNA synthesis and qRT-PCR analysis of gene expression of 19 genes were performed using the Taqman system as described previously [8] on an ABI Prism 7700 Sequence detector (Applied Biosystems Inc., Foster City, CA, USA) and the SNP RT template program or using the SYBR green system as described by Yang and others [99] was used to validate the expression of 8 genes. In both cases, the delta-delta method [100] was used to calculate relative gene expression using actin as the endogenous control. The relative transcript abundance in the controls was normalized to 1 and was used as a basis for comparison to the treatments. Plant tissue from three biological replicates was used in qRT-PCR experiments and reactions for each biological replicate were performed in duplicate (n = 6).

## Authors' contributions

SSK designed and carried out all the experiments with the assistance from SS, MM and MHR and drafted the manuscript. NNVK and MKD supervised all research and contributed to the writing and editing of the manuscript.

## Supplementary Material

Additional file 1 Transcriptional profiling: genes exhibiting more than 1.5-fold increase/decrease in transcript abundance in *ABR17 *transgenic *A. thaliana *seedlings. The data shows the genes that exhibited more than 2-fold increase/decrease in transcript abundance in two week old *ABR17 *transgenic *Arabidopsis *seedlings over two week old wild type *Arabidopsis *seedlings, both grown on normal media. Click here for file

Additional file 2 Transcriptional profiling: genes exhibiting more than 4-fold increase/decrease in transcript abundance in salt- treated wild type *A. thaliana *seedlings. The data shows the genes that exhibited more than 4-fold increase/decrease in transcript abundance in two week old salt- treated wild type *Arabidopsis *seedlings over two week old wild type *Arabidopsis *seedlings that were grown on normal media. Click here for file

Additional file 3 List of primers used in qRT-PCR. The table lists the primers used in qRT-PCR for validating microarray data Click here for file
