# Supplementary material for: Transcriptional profiling of pea ABR17 mediated changes in gene expression in Arabidopsis thaliana
Source: BMC Plant Biol. 2008 Sep 10;8:91. doi: 10.1186/1471-2229-8-91 (PMC2559843; doi:10.1186/1471-2229-8-91)
Supplement: Additional file 1 — Transcriptional profiling: genes exhibiting more than 1.5-fold increase/decrease in transcript abundance in ABR17 transgenic A. thaliana seedlings. The data shows the genes that exhibited more than 2-fold increase/decrease in transcript abundance in two week old ABR17 transgenic Arabidopsis seedlings over two week old wild type Arabidopsis seedlings, both grown on normal media. [file 1471-2229-8-91-S1.pdf]

Additional file 1. Transcriptional profiling: genes exhibiting more than 1.5-fold increase/decrease in transcript abundance in *ABR17* transgenic *A. thaliana* seedlings.

AGI [a]	Operon annotations	log2 ratio	SE	p value
At5g20230	plastocyanin-like domain containing protein	1.55	0.14	1.57E-03
At4g36060	BASIC HELIX-LOOP-HELIX (BHLH)family protein	1.49	0.19	4.33E-03
At5g44420	plant defensin protein, putative (PDF1.2a)	1.40	0.38	1.48E-02
At5g42040	hypothetical protein	1.38	0.41	2.04E-02
At4g22450	hypothetical protein	1.37	0.17	3.96E-03
At5g44430	plant defensin protein, putative (PDF1.2c)	1.35	0.40	2.03E-02
At3g45970	expansin protein family (EXPL1)	1.32	0.16	1.08E-03
At5g01920	protein kinase family	1.24	0.22	4.47E-03
At2g26010	plant defensin protein, putative (PDF1.3)	1.17	0.35	1.97E-02
At5g10040	expressed protein	1.04	0.31	2.79E-02
At1g75830	plant defensin protein, putative (PDF1.1)	1.04	0.30	1.72E-02
At2g26020	plant defensin protein, putative (PDF1.2b)	0.96	0.26	1.47E-02
At1g07135	glycine-rich protein	0.95	0.19	7.89E-03
At1g01560	mitogen-activated protein kinase (MAPK), putative (MPK11)	0.94	0.10	1.08E-02
At3g43850	hypothetical protein	0.94	0.13	5.71E-03
At2g26560	patatin, putative	0.90	0.12	6.39E-04
At5g39890	expressed protein	0.88	0.18	1.59E-02
At1g10070	tat-binding protein -related	0.88	0.06	8.70E-04
At2g22860	(AtPSK) phytosulfokine precursor 2	0.85	0.06	8.02E-04
At5g52760	heavy-metal-associated domain-containing protein	0.85	0.20	1.31E-02
At2g27080	expressed protein	0.82	0.16	1.31E-02
At1g19530	expressed protein	0.82	0.19	1.29E-02
At3g55980	expressed protein	0.82	0.19	2.41E-02
At2g47880	glutaredoxin protein family	0.82	0.22	3.43E-02
At3g62680	proline-rich protein family	0.82	0.11	4.77E-03
At4g14365	expressed protein	0.82	0.11	1.57E-03

At3g15500	No apical meristem (NAM) protein family	0.82	0.06	6.96E-04
At2g26560	patatin, putative	0.81	0.15	3.09E-03
At1g56240	F-box protein (SKP1 interacting partner 3-related)	0.80	0.12	6.07E-03
At1g08630	expressed protein	0.79	0.22	2.27E-02
At1g77120	alcohol dehydrogenase (ADH)	0.78	0.20	2.91E-02
At2g14610	pathogenesis-related protein 1 (PR-1)	0.77	0.15	3.27E-03
At1g75040	pathogenesis-related protein 5 (PR-5)	0.77	0.08	2.21E-03
At1g02660	Arabidopsis thaliana chromosome I BAC T14P4 genomic sequence	0.76	0.04	3.61E-04
At5g45340	cytochrome P450 family	0.76	0.19	2.73E-02
At2g18690	expressed protein	0.75	0.19	1.59E-02
At3g49160	pyruvate kinase -related protein	0.75	0.10	4.35E-03
At1g72940	disease resistance protein (TIR-NBS class), putative	0.75	0.08	7.77E-04
At1g18570	myb family transcription factor	0.75	0.11	6.98E-03
At1g76410	RING zinc finger protein -related	0.75	0.06	1.20E-03
A005153_01	Arabidopsis thaliana RESISTANCE TO POWDERY MILDEW8 (RPW8) locus, complete cds	0.74	0.09	4.61E-04
At2g35290	expressed protein	0.73	0.06	1.50E-03
At2g38470	WRKY family transcription factor	0.73	0.16	1.11E-02
At1g80840	WRKY family transcription factor	0.70	0.18	2.80E-02
At4g30280	xyloglucan endotransglycosylase, putative	0.70	0.12	4.58E-03
At5g54710	Arabidopsis thaliana genomic DNA, chromosome 5, TAC clone:K5F14	0.70	0.19	3.51E-02
At2g40000	nematode-resistance protein -related	0.70	0.11	1.61E-03
At2g31945	expressed protein	0.68	0.03	2.21E-04
At1g72060	expressed protein	0.68	0.10	1.11E-03
At5g46710	expressed protein	0.67	0.10	7.05E-03
At2g15890	expressed protein	0.67	0.10	1.09E-03
At1g69490	No apical meristem (NAM) protein family	0.67	0.08	4.09E-03
At4g31800	WRKY family transcription factor	0.66	0.14	5.83E-03
At1g21400	branched-chain alpha keto-acid dehydrogenase -related	0.66	0.13	7.79E-03
At3g49960	peroxidase, putative	0.66	0.03	1.30E-04
At5g47230	ethylene responsive element binding factor 5 (AtERF5)	0.66	0.19	1.67E-02

At1g07000	exocyst subunit EXO70 family	0.65	0.06	4.28E-04
At1g28330	dormancy-associated protein -related	0.65	0.12	3.02E-03
At1g02660	lipase (class 3) family	0.64	0.22	3.20E-02
At4g25920	expressed protein	0.64	0.18	3.51E-02
At1g05250	peroxidase, putative	0.64	0.08	3.80E-03
At1g02640	glycosyl hydrolase family 3	0.64	0.09	5.66E-03
At4g10270	probable wound-induced protein	0.64	0.07	2.06E-04
At4g11890	protein kinase family	0.64	0.10	8.57E-03
At4g02270	expressed protein	0.62	0.17	3.32E-02
At2g14900	gibberellin-regulated proteins -related	0.62	0.12	1.38E-02
At3g23170	expressed protein	0.62	0.04	1.05E-04
At2g19190	light repressible receptor protein kinase, putative	0.62	0.11	1.17E-02
At5g58660	oxidoreductase, 2OG-Fe(II) oxygenase family	0.62	0.12	1.48E-02
At2g18150	peroxidase, putative	0.61	0.16	1.74E-02
At1g02610	Arabidopsis thaliana chromosome I BAC T14P4 genomic sequence	0.61	0.10	8.02E-03
At5g13080	WRKY family transcription factor	0.60	0.16	3.31E-02
At2g17040	No apical meristem (NAM) protein family	0.59	0.16	3.56E-02
At4g23160	hypothetical protein	0.59	0.08	5.84E-03
At3g08720	ribosomal-protein S6 kinase (ATPK19) -related	0.59	0.07	2.90E-03
At2g41640	expressed protein	0.59	0.13	1.93E-02
At3g10040	expressed protein	0.59	0.08	5.30E-03
At2g35460	harpin-induced protein 1 family (HIN1)	0.58	0.11	6.25E-03
At5g57020	N-myristoyl transferase	-0.59	0.09	1.30E-03
At2g41650	expressed protein	-0.60	0.07	1.07E-03
At1g19150	Arabidopsis thaliana PSI type II chlorophyll a/b-binding protein (Lhca2*1) mRNA, complete cds	-0.60	0.08	5.66E-04
At1g64390	glycosyl hydrolase family 9 (endo-1,4-beta-glucanase)	-0.60	0.15	2.77E-02
At5g02230	haloacid dehalogenase-like hydrolase family	-0.60	0.07	3.64E-03
At1g62180	phosphoadenylyl-sulfate reductase (thioredoxin) (PAPS reductase), putative	-0.61	0.07	3.39E-04
At5g06530	ABC transporter family protein	-0.63	0.13	5.33E-03
At2g03090	expansin, putative (EXP15)	-0.63	0.14	2.03E-02

At5g23060	expressed protein	-0.63	0.06	1.75E-04
At4g16370	isp4 like protein	-0.64	0.07	2.52E-03
At1g23740	oxidoreductase, zinc-binding dehydrogenase family	-0.65	0.07	2.74E-04
At4g36030	armadillo repeat containing protein	-0.65	0.15	2.19E-02
At4g37980	mannitol dehydrogenase (ELI3-1), putative	-0.65	0.16	2.66E-02
At2g05100	light-harvesting chlorophyll a/b binding protein	-0.66	0.11	2.23E-03
At1g44000	expressed protein	-0.68	0.12	4.24E-03
At2g31380	salt tolerance-like protein	-0.68	0.08	8.32E-04
At1g01060	myb family transcription factor	-0.70	0.04	6.04E-05
At5g48490	protease inhibitor/seed storage/lipid transfer protein (LTP) family	-0.71	0.18	1.14E-02
At1g73870	CONSTANS B-box zinc finger family protein	-0.71	0.20	3.75E-02
At3g47420	glycerol-3-phosphate transporter (glycerol 3-phosphate permease), putative	-0.72	0.11	2.56E-03
At4g26850	expressed protein	-0.72	0.08	2.54E-04
At5g55570	hypothetical protein	-0.72	0.13	5.63E-03
At5g67370	expressed protein	-0.73	0.09	3.82E-03
At5g05250	expressed protein	-0.75	0.08	2.30E-03
At5g05270	chalcone-flavanone isomerase family	-0.75	0.06	1.16E-03
At3g02380	Zinc finger protein CONSTANS-LIKE 2 (COL2)	-0.75	0.06	2.31E-04
At5g53450	protein kinase family	-0.76	0.11	2.12E-03
At3g27690	light harvesting chlorophyll A/B binding protein, putative	-0.77	0.19	9.36E-03
At5g02120	one helix protein (OHP)	-0.90	0.09	1.66E-04
At5g48850	male sterility MS5 family	-0.99	0.17	9.96E-03
At1g56430	nicotianamine synthase, putative	-1.13	0.08	8.78E-04
At3g56980	bHLH protein family	-1.36	0.13	1.91E-03

All expression ratios are significant (α=0.05) and are in a log2 scale where fold change is ABR17/WT.

AGI [a] – *Arabidopsis* Genome Initiative SE [b] - Standard error
