# Supplementary material for: Transcriptional profiling of pea ABR17 mediated changes in gene expression in Arabidopsis thaliana
Source: BMC Plant Biol. 2008 Sep 10;8:91. doi: 10.1186/1471-2229-8-91 (PMC2559843; doi:10.1186/1471-2229-8-91)
Supplement: Additional file 2 — Transcriptional profiling: genes exhibiting more than 4-fold increase/decrease in transcript abundance in salt- treated wild type A. thaliana seedlings. The data shows the genes that exhibited more than 4-fold increase/decrease in transcript abundance in two week old salt- treated wild type Arabidopsis seedlings over two week old wild type Arabidopsis seedlings that were grown on normal media. [file 1471-2229-8-91-S2.pdf]

Additional file 3. Transcriptional profiling: genes exhibiting more than 4-fold increase/decrease in transcript abundance in salt-treated Wild type *A. thaliana* seedlings.

AGI [a]	Operon annotation	log2 scale	SE	p value
A023244_01	Arabidopsis thaliana ABA-regulated gene cluster, complete sequence	3.96	0.82	1.67E-02
A023734_01	Genomic sequence for Arabidopsis thaliana BAC F15O4 from chromosome I	3.94	0.59	1.15E-03
At1g54010	ESTs	3.90	0.28	3.55E-05
At2g38530	nonspecific lipid transfer protein 2 (LTP 2)	3.62	0.18	5.06E-06
At4g13220	expressed protein	3.59	0.35	1.60E-04
At2g02990	ribonuclease, RNS1	3.59	0.22	5.15E-04
At4g12500	protease inhibitor/seed storage/lipid transfer protein (LTP) family	3.45	0.21	1.43E-05
At2g03760	steroid sulfotransferase, putative	3.45	0.06	2.90E-08
At4g12490	protease inhibitor/seed storage/lipid transfer protein (LTP) family	3.40	0.16	4.34E-06
At2g43620	glycosyl hydrolase family 19 (chitinase)	3.38	0.24	3.57E-05
At4g04220	disease resistance protein family	3.33	0.24	3.28E-05
At3g57470	protease-related protein	3.19	0.50	1.36E-03
At4g12470	protease inhibitor/seed storage/lipid transfer protein (LTP) family	3.17	0.11	1.15E-06
At3g43180	zinc finger (C3HC4-type RING finger) protein family	3.09	0.09	4.61E-07
At5g43570	hypothetical protein	3.01	0.16	7.96E-06
At1g62420	expressed protein	2.91	0.31	6.88E-04
At3g60140	glycosyl hydrolase family 1, beta-glucosidase	2.91	0.30	6.35E-04
At1g69930	glutathione transferase, putative	2.81	0.13	2.15E-04
At3g44040	hypothetical protein	2.76	0.58	8.98E-03
At3g02240	expressed protein	2.71	0.17	1.56E-05
At4g02330	expressed protein	2.69	0.12	2.19E-04
At2g43510	trypsin inhibitor -related	2.67	0.13	4.93E-06
At5g24640	expressed protein	2.58	0.36	8.02E-04
At5g43580	hypothetical protein	2.52	0.61	8.88E-03

At4g11650	osmotin-like protein (OSM34)	2.52	0.23	1.06E-04
At3g29970	germination protein -related	2.50	0.22	8.26E-05
At5g42830	hydroxycinnamoyl benzoyltransferase-related	2.49	0.19	4.23E-05
At2g34600	expressed protein	2.48	0.55	2.06E-02
At2g16060	class 1 non-symbiotic hemoglobin (AHB1)	2.40	0.22	1.01E-04
At3g02480	expressed protein	2.40	0.24	1.85E-04
At2g13510	hypothetical protein	2.38	0.57	1.37E-02
At5g14180	expressed protein	2.36	0.30	5.10E-04
At4g25810	xyloglucan endotransglycosylase (XTR-6)	2.35	0.54	7.14E-03
At3g21720	isocitrate lyase -related	2.34	0.25	2.14E-04
At5g01330	pyruvate decarboxylase-related protein	2.34	0.19	1.15E-03
At1g10585	Genomic sequence for Arabidopsis thaliana BAC T10O24 from Chromosome 1	2.33	0.13	6.58E-05
At1g17020	oxidoreductase, 2OG-Fe(II) oxygenase family	2.32	0.11	4.15E-06
At5g36925	expressed protein	2.32	0.10	2.94E-06
At2g19800	expressed protein	2.31	0.16	3.00E-05
At1g21910	transcription factor TINY family	2.29	0.16	3.31E-05
At4g12530	protease inhibitor/seed storage/lipid transfer protein (LTP) family	2.28	0.57	2.82E-02
At2g18490	C2H2-type zinc finger protein -related	2.26	0.22	1.48E-04
At2g30840	2-oxoglutarate-dependent dioxygenase, putative	2.26	0.22	1.38E-04
At3g08860	alanine--glyoxylate aminotransferase , putative	2.25	0.16	7.38E-04
At4g12480	protease inhibitor/seed storage/lipid transfer protein (LTP) family	2.25	0.33	9.84E-04
At4g08870	arginase -related	2.25	0.15	2.56E-05
At4g36700	cupin domain-containing protein	2.22	0.65	1.86E-02
At5g36920	hypothetical protein	2.19	0.15	3.04E-05
At5g13330	AP2 domain transcription factor family	2.16	0.17	5.76E-05
At5g01920	protein kinase family	2.16	0.35	1.66E-03
At3g46270	expressed protein	2.15	0.10	4.22E-06
At1g30700	FAD-linked oxidoreductase family	2.13	0.22	6.71E-04
At5g24030	expressed protein	2.12	0.34	1.59E-03
At1g42040	hypothetical protein	2.09	0.28	6.50E-04
At5g64120	A.thaliana mRNA for peroxidase ATP15a, clone EST 151A15T7	2.08	0.20	1.44E-04

At5g19550	aspartate aminotransferase, cytoplasmic isozyme 1 (transaminase A/Asp2)	2.08	0.11	6.12E-06
At2g36780	UDP-glycosyltransferase family	2.08	0.14	2.25E-05
At4g30290	xyloglucan endotransglycosylase, putative	2.06	0.13	1.68E-05
At4g36430	peroxidase, putative	2.06	0.45	1.03E-02
At3g51860	cation exchanger, putative (CAX3)	2.05	0.24	1.09E-03
At1g35140	phosphate-induced (phi-1) protein -related	2.05	0.29	8.67E-04
At2g36770	glycosyltransferase family	2.04	0.11	3.17E-04
At4g09600	gibberellin-regulated protein GASA3 precursor	2.04	0.17	3.02E-04
At4g15910	drought-induced protein (Di21)	2.03	0.08	1.49E-06
At4g05390	ferredoxin--NADP(+) reductase (adrenodoxin reductase), putative	2.02	0.22	2.80E-04
At4g01700	glycosyl hydrolase family 19 (chitinase)	2.02	0.17	7.58E-05
At2g44370	CHP-rich zinc finger protein, putative	2.01	0.14	3.50E-05
At4g37870	Genomic sequence for Arabidopsis thaliana BAC F15O4 from chromosome I	2.01	0.13	2.46E-05
At4g16260	glycosyl hydrolase family 17	2.01	0.21	2.35E-04
At5g06330	harpin-induced protein, putative (HIN1)	2.00	0.24	1.10E-03
At5g22580	expressed protein	-2.00	0.20	1.66E-04
At4g26850	expressed protein	-2.00	0.11	9.73E-06
At3g04210	disease resistance protein (TIR-NBS class), putative	-2.00	0.20	5.13E-04
At1g25440	CONSTANS B-box zinc finger family protein	-2.02	0.16	5.55E-05
At3g08940	chlorophyll a/b-binding protein -related	-2.03	0.18	1.05E-04
At3g19320	leucine rich repeat protein family	-2.03	0.06	3.65E-07
At3g54890	light-harvesting chlorophyll a/b binding protein	-2.04	0.17	6.52E-05
At3g51750	expressed protein	-2.06	0.34	9.07E-03
At5g04550	expressed protein	-2.07	0.38	2.93E-03
At1g62360	homeobox protein -related	-2.07	0.45	1.97E-02
At3g45160	expressed protein	-2.08	0.10	5.18E-06
At1g29420	auxin-induced (indole-3-acetic acid induced) protein family	-2.08	0.16	2.12E-04
At1g14150	PsbQ domain protein family	-2.10	0.10	4.07E-06
At3g17930	expressed protein	-2.11	0.21	6.08E-04
At5g17670	expressed protein	-2.11	0.37	2.32E-03
At5g09660	malate dehydrogenase, glyoxysomal	-2.11	0.15	3.73E-05

At3g03830	auxin-induced (indole-3-acetic acid induced) protein, putative	-2.12	0.26	1.28E-03
At4g24700	expressed protein	-2.12	0.10	2.44E-04
At3g62960	glutaredoxin protein family	-2.13	0.17	5.93E-05
At2g39470	oxygen-evolving complex 25.6 kD protein, chloroplast precursor, putative	-2.14	0.28	1.48E-03
At3g04140	expressed protein	-2.14	0.12	1.09E-05
At1g78020	senescence-associated protein -related	-2.14	0.13	1.77E-05
At5g18030	auxin-induced (indole-3-acetic acid induced) protein, putative	-2.14	0.31	9.44E-04
At2g47880	glutaredoxin protein family	-2.16	0.16	3.48E-05
At2g26500	expressed protein	-2.16	0.13	1.58E-05
At5g61980	ARF GTPase-activating domain-containing protein	-2.16	0.58	1.33E-02
At1g32080	expressed protein	-2.17	0.08	1.00E-06
At5g48570	peptidylprolyl isomerase	-2.18	0.37	2.08E-03
At1g68010	glycerate dehydrogenase (NADH-dependent hydroxypyruvate reductase) (HPR)	-2.18	0.17	4.56E-05
At1g51300	Arabidopsis thaliana chromosome I BAC F11M15 genomic sequence	-2.19	0.42	6.33E-03
At5g24580	copper-binding protein family	-2.22	0.30	5.29E-03
At3g14200	DnaJ protein family	-2.23	0.35	1.41E-03
At1g12080	expressed protein	-2.23	0.39	4.52E-03
At2g45660	MADS-box protein (AGL20)	-2.24	0.12	4.70E-05
At1g61520	light-harvesting chlorophyll a/b binding protein	-2.24	0.13	1.11E-05
At4g17460	homeobox-leucine zipper protein HAT1 (HD-Zip protein 1)	-2.24	0.35	1.35E-03
At2g26020	plant defensin protein, putative (PDF1.2b)	-2.27	0.47	4.90E-03
At5g45820	CBL-interacting protein kinase 20	-2.29	0.31	7.16E-04
At3g28830	expressed protein	-2.30	0.20	8.10E-05
At5g54270	light-harvesting chlorophyll a/b binding protein, putative	-2.32	0.27	3.58E-04
At3g51895	sulfate transporter ATST1	-2.34	0.08	8.76E-06
At4g10540	Arabidopsis thaliana BAC F3H7	-2.35	0.10	1.86E-05
At5g02160	expressed protein	-2.35	0.09	1.88E-06
At3g59370	expressed protein	-2.36	0.31	5.94E-04
At3g16120	dynein light chain protein -related	-2.37	0.45	1.37E-02
At5g44780	expressed protein	-2.38	0.08	9.73E-05
At2g06230	hypothetical protein	-2.38	0.23	1.37E-04

At3g15270	squamosa promoter binding protein-related 5	-2.41	0.61	2.92E-02
At1g74310	heat shock protein 101 (HSP101)	-2.42	0.31	5.31E-04
At3g47070	expressed protein	-2.44	0.22	4.08E-04
At4g21650	Arabidopsis thaliana DNA chromosome 4, BAC clone F17L22 (ESSAII project)	-2.45	0.41	1.86E-03
At4g11320	cysteine proteinase	-2.47	0.20	5.69E-05
At1g77490	thylakoid-bound ascorbate peroxidase, putative (tAPX)	-2.51	0.19	9.75E-04
At5g44420	plant defensin protein, putative (PDF1.2a)	-2.51	0.21	7.05E-05
At1g29450	auxin-induced (indole-3-acetic acid induced) protein, putative	-2.53	0.19	4.16E-05
At1g09340	RNA-binding protein -related	-2.57	0.12	3.55E-06
At5g18080	auxin-induced (indole-3-acetic acid induced) protein, putative	-2.58	0.16	1.70E-05
At4g15460	glycine-rich protein	-2.60	0.14	4.40E-05
At4g12830	hydrolase, alpha/beta fold family	-2.60	0.18	7.77E-04
At5g42040	hypothetical protein	-2.61	0.19	3.65E-05
At2g33810	squamosa-promoter binding protein -related	-2.61	0.66	2.86E-02
At5g44430	plant defensin protein, putative (PDF1.2c)	-2.62	0.17	2.22E-05
At2g40610	expansin, putative (EXP8)	-2.66	0.08	5.28E-05
At3g15540	auxin-responsive protein IAA19 (Indoleacetic acid-induced protein 19)	-2.68	0.22	2.78E-04
At5g64770	expressed protein	-2.69	0.20	3.66E-05
At4g00755	F-box protein family	-2.70	0.25	1.18E-04
At4g26530	fructose-bisphosphate aldolase, putative	-2.72	0.25	4.07E-04
At5g22430	expressed protein	-2.73	0.50	2.78E-03
At1g29490	Arabidopsis thaliana chromosome 1 BAC F15D2 genomic sequence	-2.80	0.21	9.65E-04
At3g32130	Arabidopsis thaliana genomic DNA, chromosome 3, BAC clone: F1M23	-2.81	0.05	2.02E-08
At5g18010	auxin-induced (indole-3-acetic acid induced) protein, putative	-2.83	0.11	1.98E-06
At1g58520	ERD4 protein-related	-2.83	0.31	8.10E-04
At5g18020	auxin-induced (indole-3-acetic acid induced) protein, putative	-2.89	0.18	1.63E-05
At1g29460	auxin-induced (indole-3-acetic acid induced) protein, putative	-2.89	0.10	8.06E-07
At2g26010	plant defensin protein, putative (PDF1.3)	-2.95	0.16	7.57E-06
At5g58770	dehydrodolichyl diphosphate synthase [DEDOL-PP synthase], putative	-2.95	0.42	8.48E-04
At1g67870	glycine-rich protein	-2.97	0.18	1.52E-05
At5g39860	bHLH protein	-2.98	0.10	7.72E-05

At4g39800	myo-inositol-1-phosphate synthase	-2.99	0.17	1.06E-05
At3g09440	heat shock protein hsc70-3 (hsc70.3)	-3.03	0.22	3.37E-05
At4g21640	subtilisin-related protease	-3.04	0.31	1.79E-04
At2g15020	expressed protein	-3.04	0.42	5.34E-03
At4g28395	lipid transfer protein, putative	-3.14	0.64	1.64E-02
At2g40300	ferritin -related	-3.19	0.17	7.83E-06
At1g29510	auxin-induced (indole-3-acetic acid induced) protein, putative	-3.27	0.13	1.95E-06
At3g24500	ethylene-responsive transcriptional coactivator -related	-3.34	0.29	9.21E-05
At1g29430	auxin-induced (indole-3-acetic acid induced) protein family	-3.63	0.29	5.77E-05
At5g62080	protease inhibitor/seed storage/lipid transfer protein (LTP) family	-3.78	0.68	1.17E-02
At1g23130	Bet v I allergen family	-3.89	0.06	1.21E-08
At4g14400	Arabidopsis thaliana DNA chromosome 4, contig fragment No. 38	-3.90	0.39	1.75E-04
At1g67860	expressed protein	-4.04	0.26	1.90E-05
At5g35480	expressed protein	-4.12	0.18	3.16E-06

All expression ratios are significant (α=0.05) and are in a log2 scale where fold change is salt-treated wild type/control wild type.

AGI [a] – *Arabidopsis* Genome Initiative SE [b] - Standard error
