# Supplementary material for: Transcriptional profiling of pea ABR17 mediated changes in gene expression in Arabidopsis thaliana
Source: BMC Plant Biol. 2008 Sep 10;8:91. doi: 10.1186/1471-2229-8-91 (PMC2559843; doi:10.1186/1471-2229-8-91)
Supplement: Additional file 3 — List of primers used in qRT-PCR. The table lists the primers used in qRT-PCR for validating microarray data [file 1471-2229-8-91-S3.pdf]

Additional file 3. List of primers used in qRT-PCR

Gene	Primer pairs and probe used in real time PCR
AtExpansin	Forward; 5'-CTCCTCTGCCTCTGCTCTCTCT-3'
AT3G45970.1	Reverse; 5'-TCCGGCGAAGAAACTCGTA-3'
	Probe; 5'-TGTGCTTATGGCTCTATG-3'
AtGlycine-rich protein	Forward; 5'-GGGTGGTTCCGATTCATACG-3'
AT1G07135.1	Reverse; 5'-TGTGATCCTGCCGATCCA-3'
	Probe; 5'-TGGTAATGGCAGTCTGG-3'
AtMitogen-activated protein kinase (AtMAPK)	Forward; 5'-GGCTTGCGAGGACTAAATCAGA-3'
AT1G01560.1	Reverse; 5'-GCTCGGTACCAACGTGTAACAA-3'
	Probe; 5'-ACAGACTTCATGACAGAAT-3'
AtPlant defensin protein PDF1.2a	Forward; 5'-CCCTTATCTTCGCTGCTCTTGT-3'
AT5G44420.1	Reverse; 5'-TTCTGTGCTTCCACCATTGC-3'
	Probe; 5'-CTCTTTGCTGCTTTCGA-3'
AtIsopentenyltransferase (IPT)1	Forward; 5'-CGCCGGTGGATCTAACTCTTT-3'
AT1G68460	Reverse; 5'- AACTTTGGGTCGAATCGTTGA-3'
	Probe; 5'-TCCACGCACTCTTAG-3'

AtIPT2	Forward; 5'-TGGAATGCGCAAGTGGTTAA-3'
AT2G27760	Reverse; 5'-TTCGGTTTCTGTCTCCAGGAA-3'
	Probe; 5'-TGCTTCAGAGATCATCAG-3'
AtIPT3	Forward; 5'-TTTCCGGAGTTTGACAGGTTTT-3'
AT3G63110	Reverse; 5'-CAGTTCTTCTCTGTCTTCCACATTCA-3'
	Probe; 5'-CAGGAACGAGCAGTTC-3'
AtIPT4	Forward; 5'-TGGAGTGCCACATCACCTTCT-3'
AT4G24650	Reverse; 5'-ATTCTGCCGCTGTGACTTCTC-3'
	Probe; 5'-TGAACTAAACCCGGAGGC-3'
AtIPT5	Forward; 5'-GCCGGTGGTTCCAATTCTT-3'
AT5G19040	Reverse; 5'-CGGAAGTCAACGCAATCGT-3'
	Probe; 5'-CATCGAGGCTCTGGTC-3'
AtIPT6	Forward; 5'-GACGCTACGGCGGCAAT-3'
AT1G25410	Reverse; 5'-CCTTCTCCCTTTGCCGTACTT-3'
	Probe; 5'-ATGGCTGAGCTGAATC-3'
AtIPT7	Forward; 5'-CATTTGGGTCGACGTTTCCT-3'
NM_113267	Reverse; 5'-GCGGTCGACACGTTTTGAG-3'

	Probe; 5'-CCCGTACTTAACTCCTTT-3'
AtIPT8<br>*AT3G19160*	Forward; 5'-CCGGATCAGGCAAGTCATG-3'<br>Reverse; 5'-CGATCTCGCCAGAGAAACG-3'<br>Probe; 5'-CTCTCAATCGATCTAGCAAC-3'
AtIPT9<br>*AT5G20040*	Forward; 5'-TTGGCAGTGGCGTATTTTTG-3'<br>Reverse; 5'-AAGGCTTGGCGGTTGAAGT-3'<br>Probe; 5'-CCCGTACCTGTTACTTG-3'
AtCytokinin oxidase (CKX)1<br>*gi|20196946*	Forward; 5'-CGGGCTTGGACAGTTTGG-3'<br>Reverse; 5'-CGGTGCTGGTTCAAGAGAGAT-3'<br>Probe; 5'-ATAATCACCCGGGCACG-3'
AtCKX2<br>*AT2G19500*	Forward; 5'-CTCCCCATCATCAGCAAGGT-3'<br>Reverse; 5'-ATGAACCCGGGCAAGTAACTTA-3'<br>Probe; 5'-TGACACATTAACGAAAACA-3'
AtCKX3<br>*AT5G56970*	Forward; 5'-CCAAGGACATGAACTCGGATCT-3'<br>Reverse; 5'- TTATAATGCCGAATTGACCCAAAC-3'<br>Probe; 5'-TTCTTCGCGGTGTTAGGA-3'
AtCKX4	Forward; 5'-CCATCTCTGCCGCTTCTCA-3'

AT4G29740	Reverse; 5'-GCGCCGGGATTTTCG-3'<br>Probe; 5'-ACTTCGGTAACATAACCG-3'
AtCKX5<br>*AF303981*	Forward; 5'-TGCGGGTCGGTTCTTTATTG-3'<br>Reverse; 5'-TGAGTTGGAATCGGAGTCTCTGT-3'<br>Probe; 5'-TCGAACTCGGTCTTCA-3'
AtCKX6<br>*AT1G75450*	Forward; 5'-GATGTCGACGGCCACTTCA-3'<br>Reverse; 5'-GTCTGAGGAGACGGAGGCTAAG-3'<br>Probe; 5'-CGTCCACCCTTCC-3'
AtActin2/7<br>*AY102779*	Forward; 5'-GCCATTCAGGCCGTTCTTT-3'<br>Reverse; 5'-ATCGAGCACAATACCGGTTGT-3'<br>Probe; 5'-TCTATGCCAGTGGTCG-3'
ABA-responsive protein-related<br>*At3g02480*	Forward; 5’-GCCACTGGCCAGACTAAGGA-3'<br>Reverse; 5’- CAAGGAGTCTTGAGCTGAAGCA-3'
Xyloglucan endotransglycosylase 6<br>*At4g25810*	Forward; 5’- TTTCCTAAGAACCAGCCAATGAG-3'<br>Reverse; 5’-TTGACGAGACCACCCCTTGT-3'
basic helix-loop-helix (bHLH)<br>*At5g43650*	Forward; 5’- CCGACATCTCGGGTGATAGAA-3'<br>Reverse; 5’- CCTCAGTTCCGTGTCCTTCATAT-3'

ethylene-responsive transcription factor RAP2.6<br>*At1g43160*	Forward; 5’- TGTCCTTGGAGAGGCCAAAA-3'<br>Reverse; 5’- CATACACGTGTCGCCTTGTGT-3'
unknown protein<br>*At5g24640*	Forward; 5’- CACCACCAGTTTTTGGAGATT-3'<br>Reverse; 5’- CCGCTCACTTTCTCCGATGA-3'
ATNAC3 (*AArabidopsis NAC domain containing protein 55*)<br>*At3g15500*	Forward; 5’-TCGACGGAGGGAAGAAGAGTT-3'<br>Reverse; 5’- TTGGTTTTGGTTCCTTTTGGA-3'
ACD6 (Accelerated cell death 6)<br>*At4g14400*	Forward; 5’- CCCATGTGAAATGGCTTTTAGTC-3'<br>Reverse;5’- AGGGCCAAGGATAAAGATTGC-3'
PLAC8 domain containing protein<br>*At1g14880*	Forward; 5’- TTTGCTGTAACCTCTGTGCTTTG-3'<br>Reverse; 5’- TGCCCATCCAAGGCTCATAT-3'
